# Sensitive Sub‐THz Photodetection in Twisted Graphene with Broad Spectral Response

**DOI:** 10.1002/advs.202512851

**Published:** 2025-09-30

**Authors:** Jiaxin Wu, Meiye Hou, Shuangxing Zhu, Jun Cui, Junning Mei, Qi Sun, Yao Wang, Binghe Xie, Kenji Watanabe, Takashi Taniguchi, Zhao Liu, Qi Zhang, Xinghan Cai

**Affiliations:** ^1^ State Key Laboratory of Micro‐Nano Engineering Science Shanghai Jiao Tong University 800 Dongchuan Road Shanghai 200240 China; ^2^ School of Integrated Circuits (School of Information Science andElectronic Engineering) Shanghai Jiao Tong University Shanghai 200240 China; ^3^ National Laboratory of Solid State Microstructures and Department of Physics Nanjing University Nanjing 210093 China; ^4^ National Key Laboratory of Advanced Micro and Nano Manufacture Technology Shanghai Jiao Tong University Shanghai 200240 China; ^5^ National Institute for Materials Science Tsukuba Ibaraki 305‐0044 Japan; ^6^ Zhejiang Institute of Modern Physics Zhejiang University Hangzhou 310058 China

**Keywords:** bolometric effect, gate‐tuning, photothermoelectric effect, sub‐terahertz detection, twisted graphene superlattice

## Abstract

The exploitation of photo‐induced hot‐electron effect in graphene has enabled the advancement of ultrafast photodetectors across the visible to sub‐terahertz spectrum. However, the inherent challenges of graphene, including its zero‐bandgap, linear dispersion, and atomic‐scale thickness, impede the device's photo‐electrical conversion efficiency, resulting in a relatively moderate responsivity. Here, monolayer‐bilayer graphene into a moiré superlattice is stacked to generate gate‐tunable bandgaps and significantly modify the band structure, aiming to enhance the device's performance for sensitive broadband photodetection. The dual‐gate twisted monolayer‐bilayer graphene (TMBG) transistor exhibits consistent response patterns across the entire spectral range, with the response mechanisms identified as the photothermoelectric effect, observed without a bias voltage, and the bolometric effect, activated by applying bias. At a sub‐terahertz frequency of 0.3 THz, the transistor demonstrates exceptional performance at a low temperature of 4.5 K, with an optimized external responsivity of 16.9 A W^−1^ and a noise equivalent power of 27 fW/Hz^1/2^ and the operational temperature range can be extended up to room temperature. These findings highlight moiré graphene as a promising platform for the development of high‐performance ultra‐broadband detectors, particularly in the sub‐terahertz domain.

## Graphical abstract

A dual‐gated twisted monolayer–bilayer graphene (TMBG) phototransistor enables ultrabroadband detection spanning visible to sub‐THz regimes. Gate‐tunable moiré bands produce photothermoelectric responses at zero bias and bolometric responses under applied bias. At 0.3 THz and 4.5 K, an external responsivity of 16.9 A W^−1^ and NEP of 27 fW/Hz^1/2^ are achieved, with room‐temperature operability highlighting moiré graphene's detection potential.

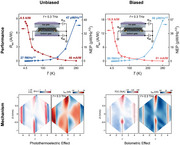

## 1 Introduction

In the rapidly evolving landscape of photodetection for the forthcoming 6G communications, there is a pressing need for devices capable of spanning the entire visible to far‐infrared spectrum, with a particular emphasis on sub‐terahertz wave detection.^[^ ^1^, ^2^ ^]^ Among the promising materials for THz sensitivity, graphene stands out due to its exceptional ability to absorb broadband electromagnetic radiation.^[^ ^3^ ^]^ Its record‐low electronic heat capacity^[^ ^4^ ^]^ and weak electron‐acoustic phonon coupling^[^ ^5^ ^]^ facilitate fast thermalization of photo‐excited charge carriers,^[^ ^6^ ^]^ leading to enhanced electron temperatures and photocurrent generation via the Seebeck or bolometric effect. Graphene‐based infrared thermal detectors exhibit tunable, ultrafast and broadband responses over a wide temperature range, outperforming commercial alternatives like the Golay cells and silicon bolometers, which often suffer from slow response times or strict operational temperature constraints.^[^ ^7^ ^]^ Nonetheless, the atomically thin nature of graphene and its zero‐gap linear‐dispersive band structure restrict the light absorption capacity^[^ ^8^ ^]^ and lead to a suboptimal temperature coefficient of the conductance (*TCC*) and Seebeck coefficient. These limitations, in turn, negatively affect the thermal‐electrical conversion efficiency and hinder the detector's external responsivity.

The twistronics approach^[^ ^9^ ^]^ emerges as a promising solution to address these limitations. Moiré graphene,^[^ ^10^, ^11^ ^]^ characterized by flat bands and superlattice‐induced bandgaps, inherits the superior properties of graphene while significantly boosting its photoresponse capabilities. These enhancements manifest in reduced thermal conductivity,^[^ ^12^ ^]^ increased Seebeck coefficient,^[^ ^13^ ^]^ and improved resistance temperature dependence,^[^ ^14^ ^]^ thus facilitating the advancement of hot‐electron photodetectors. Recent studies have demonstrated broadband photodetection in twisted graphene with varying twist angles,^[^ ^15^, ^16^, ^17^, ^18^ ^]^ extending efficient detection to the mid‐infrared regime. As about the photoresponse generated in the THz regime, magic‐angle twisted bilayer graphene, large‐twist‐angle double bilayer graphene, and twisted graphene/hBN moiré superlattices have been explored by employing diverse detection mechanisms.^[^ ^19^, ^20^, ^21^, ^22^ ^]^ While these works highlight how moiré superlattices can profoundly influence THz detection physics, achieving high‐performance photodetection with broad spectral coverage and practical operating—leveraging enhanced hot‐electron effects—remains obscured.

In this paper, we fabricate dual‐gate transistor devices by stacking monolayer and bilayer graphene with a small twisted angle ranging from 1.36°to 1.67°. The device exhibits two superlattice‐induced bandgaps above and below the flat bands, and an additional bandgap can be generated by applying a vertical displacement field. The photoresponse of the device across the visible to sub‐terahertz spectrum is then explored by varying bias and gate voltages. Without bias, a significant photoresponse is observed when the Fermi level is near the bandgap; with bias, the maximum response occurs within the bandgap region. Notably, similar response patterns are consistently observed in visible, near‐infrared, and sub‐terahertz domains, both with and without bias. Our study attributes the zero‐bias photoresponse primarily to the photothermoelectric effect, with the bolometric effect becoming dominant under bias conditions. The twisted monolayer‐bilayer graphene (TMBG) transistor demonstrates exceptional performance, particularly in the sub‐terahertz regime, with an external responsivity (*R*_ex_) exceeding 15 A W^−1^ and a calculated noise equivalent power (NEP) below 30 fW/Hz^1/2^ at a low temperature. This investigation not only elucidates the fundamental mechanisms governing TMBG photoresponse but also underscores its high‐performance potential for broad spectral coverage in advanced photodetection systems.

## 2 Results and Discussion

### 2.1 Band Structure and Transport Characteristic of TMBG

**Figure 1:**
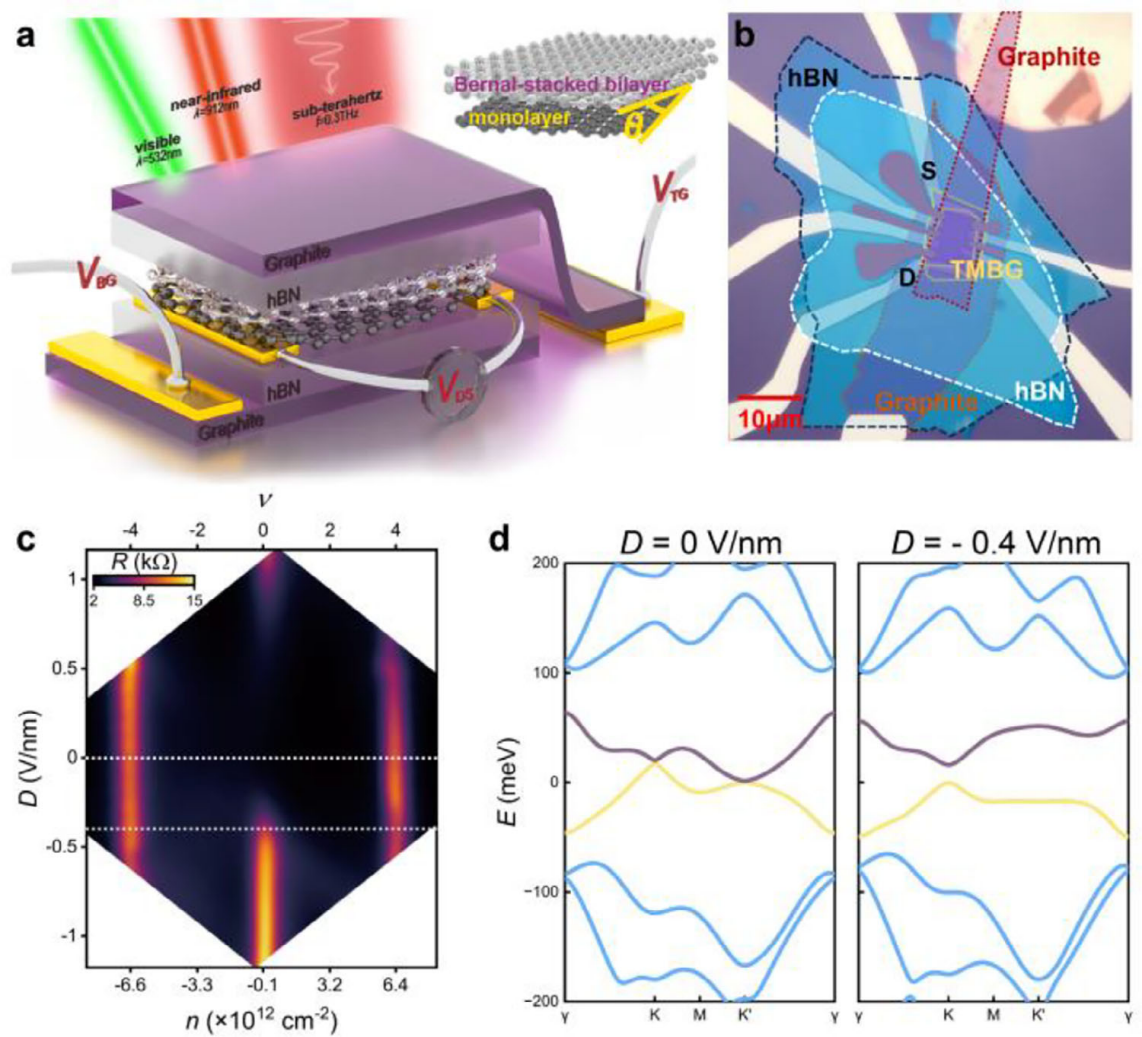
Device structure and the transport characteristic. a) Schematic of the dual‐gate TMBG transistor with measurement configuration. The stacked bilayer and monolayer graphene are encapsulated by hBN/graphite, functioning as the gate electrode and dielectric layer. The inset provides a detailed configuration of the TMBG with *θ* denoting the twist angle between two sheets of graphene layers. b) False color optical micrograph of the TMBG device, with a 10 **µm** scale bar. The source and drain electrodes used for the transport and photocurrent measurements are indicated as “S” and “D”. c) Two‐probe resistance (*R*) map as a function of carrier density (*n*) and vertical displacement field (*D*) at *T* = 4.5 K, with the corresponding band filling factor (*ν*) indicated on the top axis. d) Calculated band structures of TMBG with *θ* = 1.67° at *D* = 0 and *D* = – 0.4 V nm^−1^, corresponding to the dashed lines marked in (c). The moiré flat bands are highlighted in purple and yellow.

The active channel of our device is a twisted monolayer‐bilayer graphene with a small twist angle (*θ*), sandwiched between a top gate and a bottom gate, both composed of graphite and hexagonal boron nitride (hBN), as illustrated in **Figure** **1**a. This dual‐gate configuration allows for concurrent control of the vertical displacement field (*D*) and carrier density (*n*) in the moiré‐stacked graphene. Figure 1b presents the optical image of a representative TMBG transistor with *θ* = 1.67° (Dev. 1). The thickness of the top and bottom hBN layer is 25 and 24 nm, respectively. A comprehensive fabrication procedure is detailed in the Experimental section.

We first characterize the transport property of Dev. 1. Figure 1c displays its two‐probe resistance (*R*) map as a function of *n* and *D* at a temperature of *T* = 4.5 K, with an AC bias current of 50 nA applied between the source and drain electrodes. Without a displacement field, two resistance peaks are observed at *n* ≈ 6.4 × 10^12^ and −6.6 × 10^12^cm^−2^, respectively, indicating the formation of superlattice‐induced bandgaps above and below the flat moiré bands^[^ ^23^ ^]^ (see Figure 1d left). These bandgaps appear to be relatively insensitive to the displacement field magnitude. Given the four‐fold valley and spin degeneracy in the lowest moiré bands, a full occupation of one moiré band necessitates four electrons or holes per cell. The band filling factor (*ν*), indicated on the top axis of Figure 1c, enables the estimation of the twist angle. A distinct resistance peak emerges near the charge neutrality point when a substantial *D* is applied. The experimental resistance map of our TMBG transistor aligns with the theoretically derived band structures (Figure 1d), which reveals the presence of gaps both above and below the moiré flat bands, and an additional gap opens in the vicinity of the charge neutrality point at an appreciably high value of the displacement field (*D* = −0.4 V nm^−1^, Figure 1d right). From the calculated band structures (refer to Figures S1,S2,S4, and S5, Supporting Information), the gap at the charge neutrality point under a displacement field of *D* = −1.1 V nm^−1^ is estimated to be 29.4 meV, while the gap at the flat‐band filling position (*D* = 0 V nm^−1^) reaches 39.6 meV. These compare well with our transport measurements – Arrhenius analysis yields experimental gaps of ≈11.4 meV at charge neutrality and ≈27.2 meV at the superlattice‐induced gap (see Figure S3, Supporting Information for detailed discussion).

### 2.2 Ultra‐Broadband Photoresponse of the TMBG Transistor

**Figure 2:**
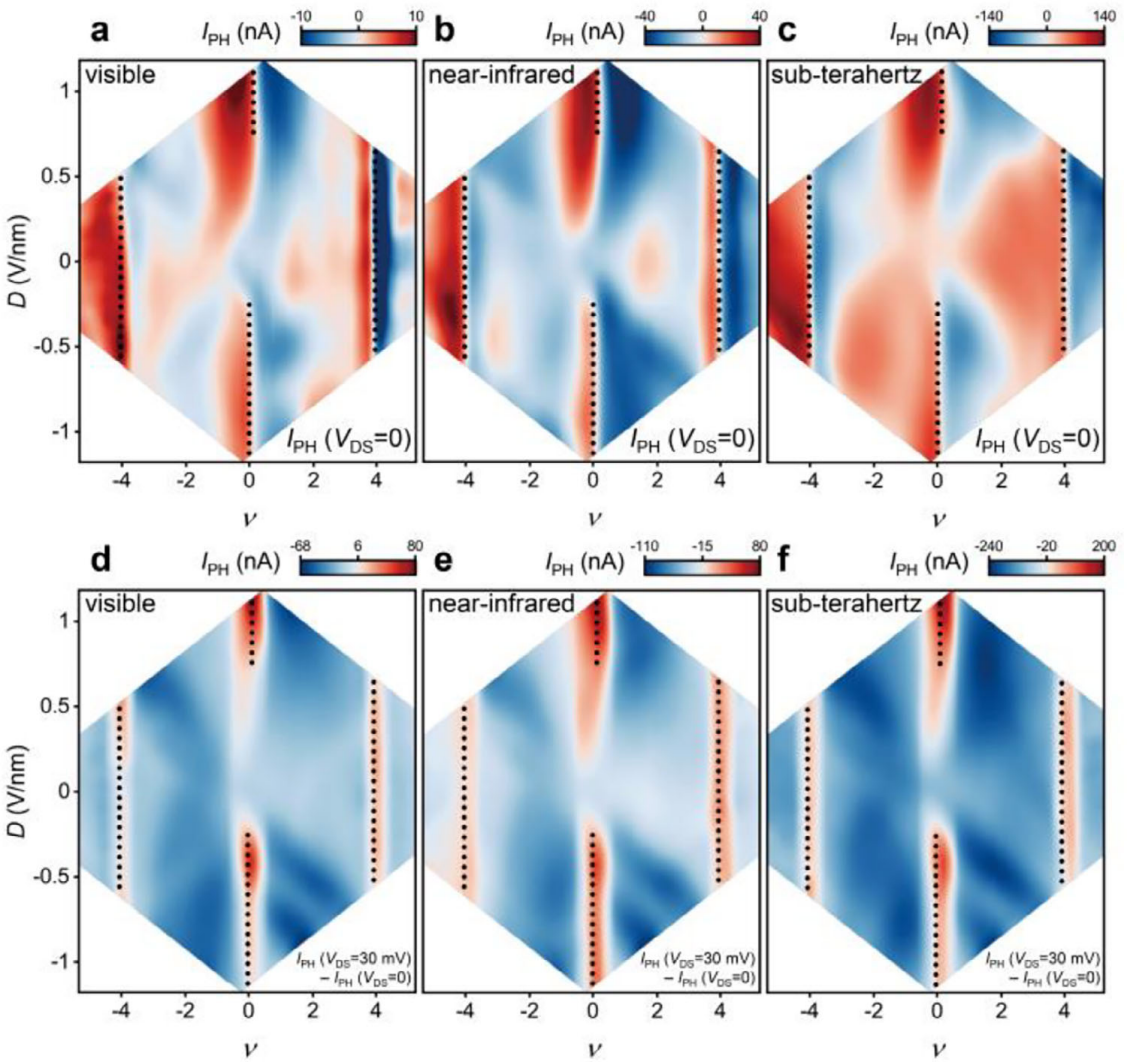
Photoresponse of the TMBG transistor from visible to sub‐terahertz spectrum. a–c) Photocurrent maps as a function of *ν* and *D* at *V*_DS_ = 0, with light illumination at wavelengths of 532 nm with a power of 8 µW (a), 912 nm with a power of 12 µW (b), and a frequency of 0.3 THz with a power density of 743 µW mm^‐^^2^ (c). d–f) Differential photocurrent maps as a function of *ν* and *D* obtained by subtracting the response at zero bias from that at *V*_DS_ = 30 mV, for corresponding wavelengths of 532 nm (d), 912 nm (e), and a frequency of 0.3 THz (f). The black dashed traces in (a–f) indicate high resistance regions referring to bandgaps, as determined by transport characteristic. All measurements are performed at a temperature of *T* = 4.5K.

Next, we examine the broadband photoresponse of the TMBG transistor by sequentially exposing the device to visible (*λ* = 532 nm, beam diameter ≈ 1.5 µm), near‐infrared (*λ* = 912 nm, beam diameter ≈ 2 µm), and sub‐terahertz (*f* = 0.3 THz, beam diameter ≈ 6 mm) radiation. **Figure** **2**a–c displays the short‐circuit (*V*_DS_ = 0) photocurrent map, which varies with the band filling factor and the vertical displacement field, both modulated by the top and bottom gate voltages. Pronounced photocurrent is observed across all wavelengths when the Fermi level aligns with the superlattice bandgap (near *ν* = ±4), with polarity reversal occurring at the black dashed lines, serving as visual guides for the bandgap locations. Close to the charge neutrality (*ν* = 0), the photocurrent magnitude increases gradually as the displacement field opens the bandgap between flat bands and the signal flips its sign when the Fermi level crosses the charge neutral point. Despite minor variations potentially due to differences in beam size and device inhomogeneity, the consistent primary features in the three photocurrent maps imply a shared physical mechanism underlying the zero‐bias photoresponse across various electromagnetic excitation wavelengths. To ensure accuracy, the sub‐terahertz incident power was calibrated via beam profiling and direct power measurements (see Figure S6, Supporting Information), further supporting the reliability of the observed trends.

The impact of a bias voltage on the photocurrent is then explored. Figure 2d–f presents differential photocurrent maps for excitation at different wavelengths, derived by subtracting the photocurrent at a DC bias of *V*_DS_ = 30 mV from that without bias, for the same top and bottom gate voltages applied. Consistent responses are evident across visible, near‐infrared, and sub‐terahertz ranges: Positive photocurrent peaks appear at the Dirac point with substantial displacement fields and at superlattice‐induced bandgaps, while notable negative photocurrent is observed when the Fermi level is tuned to the flat band region between the Dirac point and the bandgaps (*ν* = 0–4).

The photoresponse of the TMBG transistor in the mid‐infrared range is also demonstrated, exhibiting characteristics consistent with those observed in the near‐infrared when illuminated by a 10.6 µm laser, as described in Figures S7 and S8 (Supporting Information). The distinct photocurrent patterns with and without a bias indicate multiple photoresponse mechanisms in the dual‐gate TMBG device, showcasing its tunable photodetector functionality, capable of ultra‐broadband detection spanning the visible to sub‐terahertz spectrum.

### 2.3 Physical Mechanism of the Broadband Photoresponse

**Figure 3:**
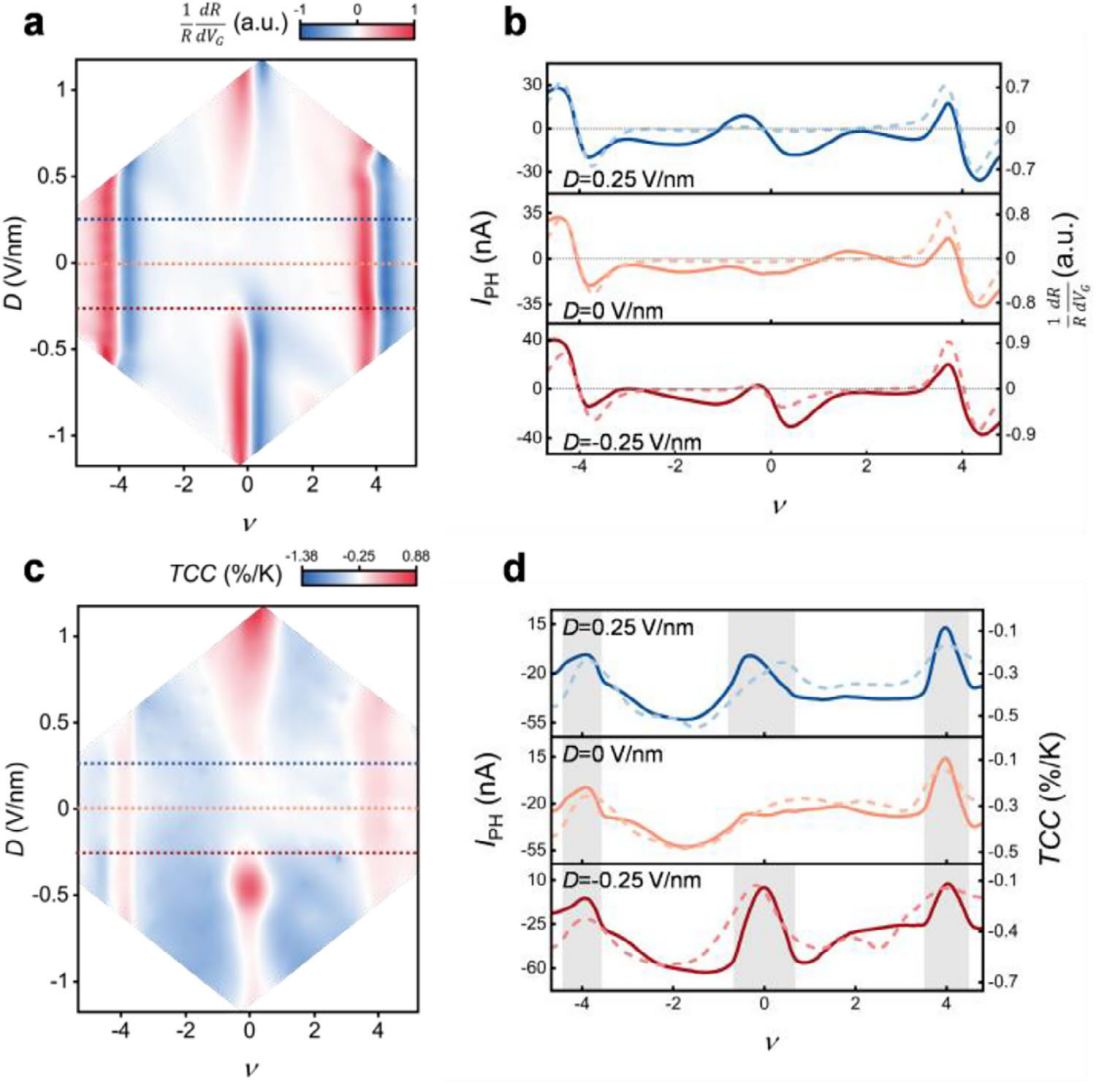
Physical mechanism of the photoresponse in the TMBG transistor. a) Calculated $\frac{1}{R}\frac{dR}{dV_{G}}$ as a function of *ν* and *D*, derived from the transport data using the Mott formula. The blue, orange, and red dashed lines correspond to the displacement fields in (b). b) Photocurrent line cuts (solid lines, left axis) at zero bias with 912 nm illumination (Figure 2b) at displacement fields of *D* = 0.25 V nm^−1^, *D* = 0, and *D* = −0.25 V nm^−1^, overlaid with the corresponding $\frac{1}{R}\frac{dR}{dV_{G}}$ profiles (dashed lines, right axis). c) Calculated temperature coefficient of the conductance (*TCC*) as a function of *ν* and *D* at 4.5 K, derived from temperature‐dependent conductance measurements. The blue, orange, and red dashed lines correspond to the displacement fields in (d). d) Photocurrent line cuts (solid lines, left axis) from differential photocurrent maps with 912 nm light illumination (Figure 2e), overlaid with the corresponding *TCC* profiles (dashed lines, right axis). Peaks are emphasized against a grey background.

To elucidate the photocurrent generation mechanism in the TMBG device, we compare the photoresponse with the electrical transport data. In the photothermoelectric (PTE) picture, photo‐excited hot carriers diffuse along a temperature gradient, and non‐uniform light illumination or device asymmetry drives a net current. This process can be described by the thermoelectric photocurrent expression:

(1) $$I_{\mathrm{PTE}}=-\frac{1}{R}\int S\nabla T_{e}dx$$

where ∇*T*_e_ is the electron temperature gradient, *R* is the two‐probe resistance and *S* is the Seebeck coefficient, given by the Mott formula:^[^ ^24^ ^]^

(2) $$S=-\frac{\pi^{2}k_{B}^{2}T_{e}}{3e}\frac{1}{G}\frac{dG}{dE_{F}}$$

with k_B_, e, *G*, and *E*_F_ denote to the Boltzmann constant, elementary charge, device's conductance, and the Fermi energy, respectively. Simplifying Equation (2) reveals that the Seebeck coefficient is proportional to $\frac{1}{R}\frac{dR}{dV_{G}}$, which can be derived from the transport measurement. In **Figure** **3**a, $\frac{1}{R}\frac{dR}{dV_{G}}$ is plotted as a function of *ν* and *D*, which qualitatively reproduces the profile of the short‐circuit photocurrent maps (Figure 2a–c), substantiating the photothermoelectric effect as the primary photoresponse mechanism at zero bias (see Figures S9 and S10, Supporting Information for the locally excited PTE response and its sign analysis). Figure 3b further compares photocurrent line cuts under 912 nm illumination (solid lines, left axis) with the corresponding $\frac{1}{R}\frac{dR}{dV_{G}}$ profiles (dashed lines, right axis) at *D* = 0.25 V nm^−1^, *D* = 0 and *D* = −0.25 V nm^−1^. The photocurrent curves exhibit an “S”‐shaped dependence on *ν*, reversing polarity across the bandgaps—similar to the gate dependence of the Seebeck coefficient in graphene.^[^ ^25^ ^]^ Additionally, the reversal of the Seebeck signal trend upon masking one graphene‐metal contact confirms that the net photocurrent stems from asymmetry the two contacts (refer to Figure S11, Supporting Information for further details).

In the biased condition, the photocurrent behavior can be explained by the photo‐bolometric effect. The bolometric current, *I*_BOL_, is governed by the equation:

(3) $$I_{\mathrm{BOL}}=V_{\mathrm{DS}}\;\cdot \Delta G=V_{\mathrm{DS}}\cdot G\cdot TCC\cdot \Delta T$$

where $TCC=\frac{1}{G}\;\frac{\Delta G}{\Delta T}$ represents the temperature coefficient of conductance. To determine *TCC*, we compare conductance data measured at *T* = 32K — extracted from the elevated‐temperature transport maps in Figure S12a (Supporting Information) — with the *T* = 4.5K dataset in Figure 1c. The *TCC* map at *T* = 5K exhibits pronounced features at both the superlattice‐induced bandgaps and the flat‐band regions. Figure 3d compares photocurrent line cuts under a bias of *V*_DS_ = 30 mV (solid lines, left axis) with the corresponding *TCC* profiles (dashed lines, right axis) for *D* = 0.25 V nm^−1^, *D* = 0, and *D* = −0.25 V nm^−1^. Positive photocurrent peaks occur when the Fermi level lies within the bandgaps, while substantial negative responses appear in the flat‐band regions. This polarity reversal is consistent with the bolometric mechanism, in which the device behaves as an insulator or a metal depending on the Fermi level position, resulting in opposite signs of the temperature coefficient of conductance. The close correspondence between the *TCC* distribution and the biased photocurrent patterns confirms that the bolometric effect dominates the photoresponse under an applied bias (refer to Figure S13, Supporting Information for further details).

Consequently, our analysis suggests that the broadband photoresponse mechanism in the TMBG device combines both photothermoelectric and bolometric effects. The photothermoelectric effect prevails under zero‐bias conditions, while the bolometric effect becomes significant in biased scenarios, consistent with spatial and temperature‐dependent photocurrent measurements (see Figures S14–S17, Supporting Information).

### 2.4 Sensitive Sub‐Terahertz Photodetection

**Figure 4:**
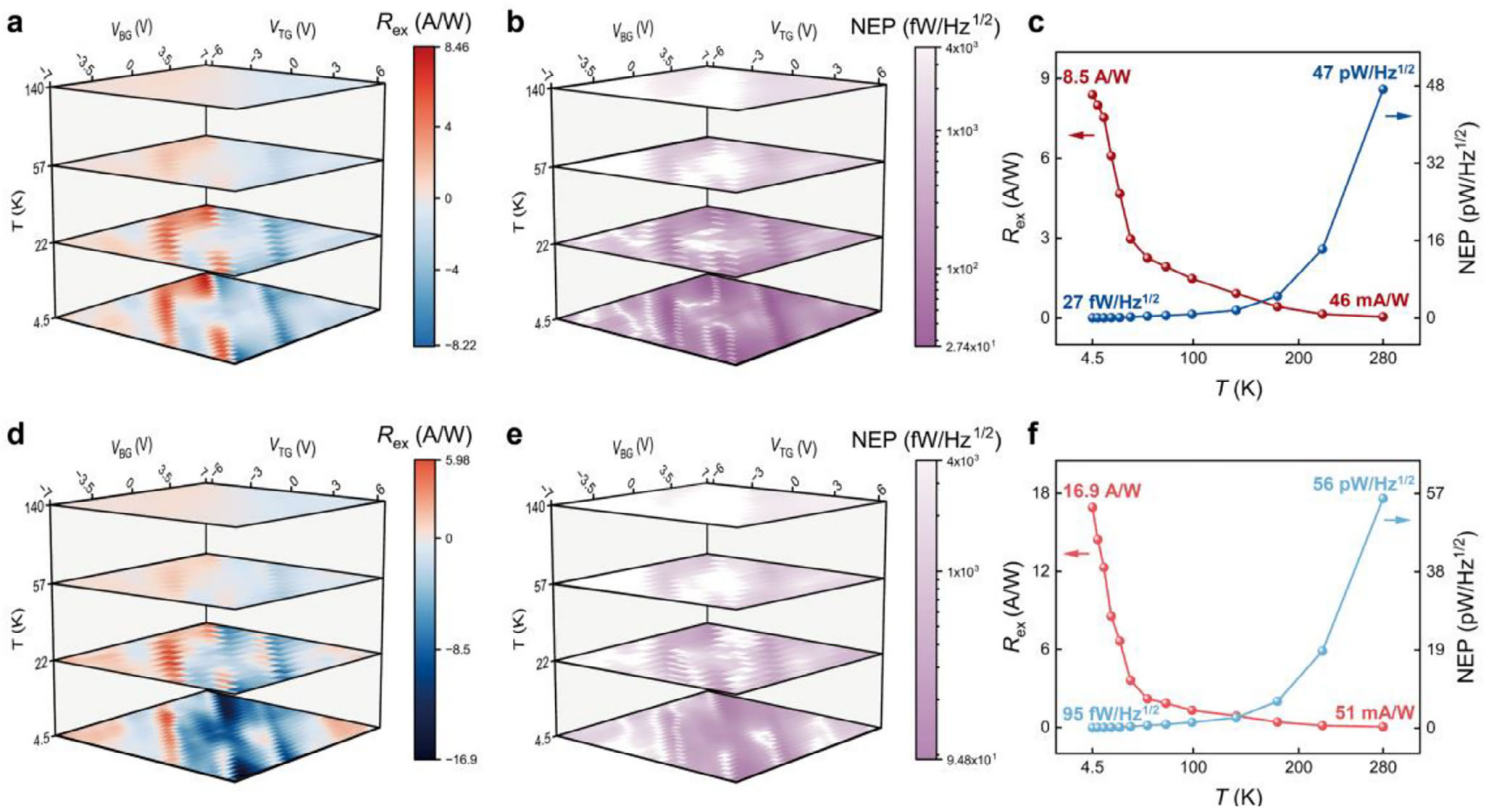
Superior sub‐terahertz performance of the TMBG photodetector. a,d) Evolution of external photoresponsivity (*R*_ex_) map with temperature at a bias voltage of *V*_DS_ = 0 (a) and 30 mV (d), respectively. The frequency and power density of the incident sub‐terahertz laser is *f* = 0.3 THz and *P* = 1.49 mW mm^−2^. b,e) Evolution of calculated noise equivalent power (NEP) map with temperature at a bias voltage of *V*_DS_ = 0 (b) and 30 mV (e), respectively. c,f) The optimized *R*_ex_ and NEP as a function of the temperature for *V*_DS_ = 0 (c) and 30 mV (f), respectively.

The exceptional ability of TMBG to sense electromagnetic radiation across a broad spectrum makes it an ideal choice for developing high‐performance terahertz detectors with superior responsivity. We fabricated multiple TMBG devices with varying twist angles to evaluate their terahertz performance, with reproducibility verified by consistent measurements in a second device (*θ* = 1.36°; Figures S18–S21, Supporting Information). **Figure** **4** presents the responsivity and noise‐equivalent power (NEP) of Dev. 2 in the sub‐terahertz range at different temperatures. We first demonstrate the external responsivity (*R*_ex_) of the photothermoelectric signal, defined as the ratio of photocurrent to incident illumination power on the TMBG, without bias voltage (Figure 4a). At 4.5 K, the photocurrent changes sign near the bandgap, and the optimized photoresponse reaches a maximum of 8.46 A W^−1^. As the temperature rises, the density of states no longer exhibits sharp features due to thermal band broadening, resulting in a decrease in the Seebeck coefficient and consequently a reduction in the photocurrent. Assuming no dark current (no shot noise) and a high laser modulation frequency (negligible 1/*f* noise), the primary source of noise in unbiased TMBG photodetectors is Johnson noise, calculated as $N_{\mathrm{johnson}}=\sqrt{\frac{4k_{B}T}{R}}\;$. Figure 4b demonstrates the derived NEP ($\frac{N_{\mathrm{johnson}}}{R_{\mathrm{ex}}}$) as a function of top and bottom gate voltages at various temperatures, with a minimum NEP of 27 fW/Hz^1/2^ at *T* = 4.5K. Figure 4c further presents the optimized *R*_ex_ and NEP values across a broader temperature range. The device remains functional at room temperature, exhibiting an external responsivity of 46 mA W^−1^ and a calculated NEP of 47 pW/Hz^1/2^. Time‐domain measurements further reveal that the sub‐THz photocurrent exhibits a fast response speed (see Figure S22, Supporting Information).

We further investigate the device's performance with an applied bias of *V*_DS_ = 30 mV (Figure 4d–f), where both photothermoelectric and bolometric effects contribute to the photocurrent signal. At *T* = 4.5 K, a substantial negative response is observed in the flat‐band regions, with the maximum external responsivity peaking at 16.9 A W^−1^. As the temperature rises, the bolometric signal rapidly diminishes, and above *T* = 57 K, the response patterns show negligible differences with or without bias, indicating a near‐zero bolometric response. Since an applied bias introduces a dark current, it is crucial to account for both Johnson noise and shot noise, with the latter calculated as $N_{\mathrm{shot}}=\sqrt{2eI_{\mathrm{dark}}}\;$. The total noise is determined by $N_{\mathrm{total}}=\sqrt{N_{\mathrm{johnson}}^{2}+N_{\mathrm{shot}}^{2}}\;$. Figure 4e illustrates the evolution of the NEP ($\frac{N_{\mathrm{total}}}{R_{\mathrm{ex}}}$) map as a function of top and bottom gate voltages across different temperatures. At low temperatures, the minimum NEP reaches 95 fW/Hz^1/2^, which is slightly higher than that under the zero‐bias condition due to the presence of the shot noise. Figure 4f summarizes the optimized *R*_ex_ and NEP from 4.5 to 280K. The device's performance exhibits significant changes in the low temperature regime, with an external responsivity of 56 mA W^−1^ and a NEP of 51 pW/Hz^1/2^ observed at room temperature.

Our TMBG photodetector exhibits a substantial enhancement in external responsivity at low temperatures compared to previously reported monolayer graphene (MLG)^[^ ^26^ ^]^ and bilayer graphene (BLG)^[^ ^27^ ^]^ devices with the same dual‐gate configuration. We note that optimized responsivity and NEP values occur either near superlattice‐induced bandgaps or close to the charge neutrality point under large displacement fields. While the charge neutrality point gap is smaller, it generates a larger photocurrent due to the sharp density‐of‐states variations and reduced Fermi velocity, which enhances the Seebeck coefficient. In contrast, the lowest NEP is found near superlattice‐induced gaps, where lower device resistance minimizes Johnson noise. This distinction highlights that responsivity and NEP depend not only on gap size but also on detailed band‐structure features and transport asymmetries. The optimized NEP is impressively low, outperforming contemporary bolometers on the market (refer to Table S2, Supporting Information for a comprehensive comparison). Although the NEP increases at room temperature, our detector's performance metrics still display a notable advantage, surpassing recent graphene‐based terahertz detectors^[^ ^28^, ^29^, ^30^ ^]^ (refer to Tables S2 and S3, Supporting Information).

We notice that several recent studies have observed interband‐transition‐induced bulk photovoltaic effects in twist‐aligned graphene devices with comparable bandgap sizes.^[^ ^18^, ^21^, ^22^ ^]^ In contrast, our dual‐gated TMBG devices demonstrate a dominant hot‐carrier response across a broad spectral range (sub‐THz to visible). This distinct behavior can be attributed to: 1) twist‐angle inhomogeneity (Figure S21, Supporting Information), which disrupts coherent symmetry‐breaking photocurrents, and 2) our bottom‐contact geometry that facilitates pn‐junction formation through enhanced doping compared to the 1D edge contacts in Ref. [18] These combined factors maintain the thermal hot‐carrier pathway as the predominant detection mechanism in our TMBG devices, even when excitation energies significantly exceed the moiré gap energies. Other recent studies have identified diverse photocurrent generation mechanisms across other graphene moiré systems, including: the Berry‐geometry‐driven photogalvanic effect in magic‐angle twisted bilayer graphene, the photoconductive responses from multi‐gap moiré bands in large‐twist‐angle double bilayer graphene, and the dual mechanisms (intraband plasma‐wave rectification and interband photovoltaic effects) in twisted graphene/hBN heterostructures.^[^ ^20^, ^21^, ^22^ ^]^ Crucially, some of these mechanisms exhibit strong dependence on the excitation frequency, which is essential for designing moiré‐based photodetectors and optimizing their performance. Nonetheless, our dual‐gated TMBG platform offers some unique benefits, as its hybrid moiré band structure merges robust superlattice‐induced gaps with a displacement‐field‐tunable charge neutrality gap, allowing seamless switching between photothermoelectric and bolometric modes in a single device. This dual‐mode operation delivers high responsivity and low noise‐equivalent power across visible to the sub‐THz frequencies in a cryogenic regime, while retaining functionality up to room temperature. In addition, the dual‐gate design allows independent control of carrier density and displacement field, providing superior operational precision compared to single‐gated devices. There is extensive potential for further improvement in the TMBG photodetector, particularly by controlling the fabrication process to introduce deliberate asymmetry, like employing local split gates ^[^ ^25^ ^]^or different metal contacts,^[^ ^31^ ^]^ which can enhance the zero‐bias photothermoelectric response and NEP. For bolometric response, increasing the bias voltage beyond 30 mV or trying to reduce the contact resistance would amplify the signal, thereby boosting responsivity according to Equation (3). In terms of large‐scale manufacturing, recent advancements in wafer‐scale moiré graphene fabrication^[^ ^32^, ^33^ ^]^ promise to significantly enhance the effectiveness of TMBG‐based terahertz optoelectronic devices through integration methods compatible with conventional silicon technology.

Moreover, we point out that the photothermoelectric and bolometric responses in TMBG device exhibit remarkable tunability through bias voltage, enabling a diverse range of detection strategies for hybrid applications. In optical communication systems^[^ ^34^ ^]^ or in medical imaging^[^ ^35^ ^]^ where the focus is on the signal strength or on the luminosity of images, applying a large bias voltage to employ the bolometric effect augments the device's response intensity. In other scenarios such as astronomical observation^[^ ^36^ ^]^ and environmental monitoring,^[^ ^37^ ^]^ where detecting weak illumination with minimized signal‐to‐noise ratio is critical, operating the device in the photothermoelectric mode without bias is preferable to optimize the NEP. Future extensions of this work—such as multi‐element TMBG arrays with local gates or integration of nonvolatile gating materials (e.g., ferroelectrics, ion gels)—could enable stateful optical memory and photo‐programmable logic, building on recent advances in reconfigurable optoelectronic systems.^[^ ^38^, ^39^ ^]^

## 3 Conclusion

In summary, dual‐gate TMBG transistors enable ultra‐broadband detection across a spectrum from visible to sub‐terahertz frequencies in a wide temperature range. The underlying mechanisms of the photothermoelectric effect in unbiased conditions and the bolometric effect under bias are unraveled through the analysis of gate‐dependent photocurrent measurements, which are compared with theoretical calculations derived from transport data. The device demonstrates superior performance, with an external responsivity of 16.9 A W^−1^ and an NEP of 27 fW/Hz^1/2^ at low temperature. These outstanding metrics, along with the tunable photoresponse, make TMBG transistors a highly versatile solution for a diverse array of photodetection applications.

## 4 Experimental Section

### Device Fabrication

Graphene and hBN nano‐flakes were mechanically exfoliated onto SiO_2_/Si substrates. The determination of graphene layer number was conducted through optical contrast and Raman spectroscopy, while the thickness of the hBN, employed as the top and bottom dielectric layers, was ascertained via atomic force microscopy (AFM). All van der Waals sheets were assembled using the standard dry‐transfer techniques^[^ ^40^ ^]^ with polycarbonate (PC)/polydimethyl siloxane (PDMS) stamps. To begin with, hBN and graphite were transferred to a 285 nm SiO_2_/Si substrate to establish a bottom gate. Ti/Au (10 nm/25 nm) were subsequently deposited on the bottom gate stack, following the electron beam lithography (EBL) process. These selected flakes were then sequentially transferred and stacked onto the metal electrodes. The twisted monolayer‐bilayer configuration was prepared via the “cut‐and‐stack” method:^[^ ^41^ ^]^ A graphene sample, comprising a flake with distinct areas of single‐layer and double‐layer domains, was precisely bisected along its boundary using a hard diamond‐coated atomic force microscopy (AFM) tip. The resulting single‐layer and double‐layer pieces were then twist‐aligned and stacked together. Finally, plasma generated from a mixture of CHF_3_ and O_2_ gases was utilized to etch the non‐stacked regions of the graphene layers.

### Electrical Transport Measurements

Low‐temperature transport measurements were conducted within a closed‐cycle optical cryostat (Montana Instruments Corp.; S50). The two‐probe resistance was measured using a lock‐in amplifier, which served as the AC source. A current‐limiting circuit was employed, by connecting the sample in series with a very high‐resistance resistor, and the sample voltage was measured using the same lock‐in amplifier. This setup was linked to a multifunction I/O device (National Instruments) to facilitate data acquisition and recording.

To enable precise control of carrier density (*n*) and vertical displacement field (*D*) simultaneously, a dual‐gate geometry is employed in which the top‐gate (*V*_TG_) and bottom‐gate (*V*_BG_) voltages can be tuned independently. Following the standard electrostatic model for dual‐gated graphene systems, the two quantities are defined as:

(4) $$n=\frac{C_{\mathrm{TG}}V_{\mathrm{TG}}-C_{\mathrm{BG}}V_{\mathrm{BG}}}{e},\;D\;=\frac{C_{\mathrm{TG}}V_{\mathrm{TG}}-C_{\mathrm{BG}}V_{\mathrm{BG}}}{2\varepsilon_{0}}$$

where *C*_TG_ and *C*_BG_ are the sheet capacitances of the top and bottom gate respectively, *e* is the elementary charge, and ε_0_ is the dielectric constant. his quantitative framework provides a one‐to‐one mapping between gate voltage pairs (*V*_TG_, *V*_BG_) and corresponding (*n*, *D*) coordinates, thereby enabling fully independent control over both carrier density and displacement field.

### Broadband Photoresponse Measurements

A variety of laser sources were employed to stimulate the photocurrent response in the device. For visible to near‐infrared illumination, a focused continuous‐wave laser with a wavelength of 532 nm (diameter ≈ 1.5 µm; Changchun New Industries Optoelectronics Tech. Co., Ltd. (CNI); MLL‐III‐532) or 912 nm (diameter ≈ 2 µm; Changchun New Industries Optoelectronics Tech. Co., Ltd. (CNI); MIL‐H‐912) was employed, modulated using a mechanical chopper operating at 277 Hz. In the sub‐terahertz regime (*f* = 0.3 THz), a large diameter source (≈ 6 mm; Terasense, Tunable Sub‐Terahertz Wave Source) was driven by a TTL square wave, generated by a multifunction I/O device (National Instruments) at a frequency of 1617 Hz. The photocurrent signal was captured using a current preamplifier (DL Instruments; Model 1211) and a lock‐in amplifier (Stanford Research Systems; Model SR830), which is synchronized with the modulation frequency of the incident light source. TTL chopping introduces additional noise to the lock‐in amplifier, which is significantly greater than that caused by mechanical chopping.

## Conflict of Interest

The authors declare no conflict of interest.

## Author Contributions

J.W. and M.H. contributed equally to this work. X.C. and Q.Z. conceived the idea and led the research direction. Q.Z. contributed equipment support for the sub‐terahertz experiment. Z.L. provided theoretical support with the single‐electron model. J.W. fabricated the devices and conducted the experimental testing. M.H. provided support in building the optical setup. S.Z., J.C., J.M., Q.S., Y.W., and B.X. assisted in various stages of the experiment and data analysis. K.W. and T.T. supplied the high‐quality hBN crystals for device fabrication. X.C. and J.W. wrote the manuscript with contributions from all authors.

## Supporting information



Supporting Information

## Data Availability

Research data are not shared.
