# Supplementary material for: Sensitive Sub‐THz Photodetection in Twisted Graphene with Broad Spectral Response
Source: Adv Sci (Weinh). 2025 Sep 30;12(46):e12851. doi: 10.1002/advs.202512851 (PMC12697868; doi:10.1002/advs.202512851)
Supplement: Supplementary file 1 — Supporting Information [file ADVS-12-e12851-s001.docx]

Supplementary Information for

**Sensitive sub-THz Photodetection in Twisted Graphene with Broad Spectral Response**

*Jiaxin Wu, Meiye Hou,* *Shuangxing Zhu, Jun Cui, Junning Mei, Qi Sun, Yao Wang, Binghe Xie, Kenji Watanabe, Takashi Taniguchi, Zhao Liu**,^*^ Qi Zhang,^*^ Xinghan Cai,^*^*

Jiaxin Wu, Shuangxing Zhu, Binghe Xie

State Key Laboratory of Micro-Nano Engineering Science, Shanghai Jiao Tong University, 800 Dongchuan Road, Shanghai 200240, China;

Department of Micro/Nano Electronics, School of Integrated Circuits, Shanghai Jiao Tong University, 200240 Shanghai, China;

Meiye Hou, Jun Cui, Qi Zhang

National Laboratory of Solid State Microstructures and Department of Physics, Nanjing University, 210093 Nanjing, China;

Email: zhangqi@nju.edu.cn

Junning Mei, Qi Sun, Yao Wang

Department of Micro/Nano Electronics, School of Integrated Circuits, Shanghai Jiao Tong University, 200240 Shanghai, China;

National Key Laboratory of Advanced Micro and Nano Manufacture Technology, Shanghai Jiao Tong University, 200240 Shanghai, China;

Kenji Watanabe, Takashi Taniguchi

National Institute for Materials Science, Tsukuba, Ibaraki 305-0044, Japan;

Zhao Liu

Zhejiang Institute of Modern Physics, Zhejiang University, 310058 Hangzhou, China.

Email: zhaol@zju.edu.cn

Xinghan Cai

State Key Laboratory of Micro-Nano Engineering Science, Shanghai Jiao Tong University, 800 Dongchuan Road, Shanghai 200240, China;

Department of Micro/Nano Electronics, School of Integrated Circuits, Shanghai Jiao Tong University, 200240 Shanghai, China;

National Key Laboratory of Advanced Micro and Nano Manufacture Technology, Shanghai Jiao Tong University, 200240 Shanghai, China;

Email: xhcai@sjtu.edu.cn

Author 1 and Author 2 contributed equally to this work.

List of contents:

Note 1. Single-electron continuum model of TMBG.

Note 2. Band structure of TMBG with a twist angle of 1.67°.

Note 3. Twist angle dependence of the band structure.

Note 4. Determination of sub-terahertz incident power.

Note 5. Mid-infrared photoresponse of Dev. 3.

Note 6. Locally excited photothermoelectric response.

Note 7. Temperature dependent transport properties of Dev.1.

Note 8. Additional characterization of the bolometric response in Dev. 1.

Note 9. Comparison between the photothermoelectric and bolometric response in Dev. 1.

Note 10. Temperature dependence of the sub-terahertz photoresponse in Dev.1.

Note 11. Band structure of TMBG with a twist angle of 1.36°.

Note 12. Ultra-broadband photoresponse in Dev.2.

Note 13: Twist-angle inhomogeneity of Dev.2.

Note 14: Response Speed Benchmarking.

Note 1: Single-electron continuum model of TMBG.

We use an extension of the Bistritzer-MacDonald continuum model^[1-3]^ to calculate the low-energy band structure of TMBG. Under the approximation of continuum model, the two valleys of TMBG are decoupled and related by time-reversal conjugate, so we focus on a single valley + in what follows.

We assume that the top (t) sheet is monolayer graphene and the bottom (b) sheet is bilayer graphene. In the moiré Brillouin zone (MBZ) of valley +, the Dirac points of the top and bottom sheets are located at $\boldsymbol{K}_{+}^{t}=R_{\theta/2}\boldsymbol{K}_{+}$ and $\boldsymbol{K}_{+}^{b}=R_{-\theta/2}\boldsymbol{K}_{+}$, respectively (Figure S1), where $R_{\theta}$ is the counter-clockwise rotation by angle $\theta$, and $\boldsymbol{K}_{+}$ is the Dirac point without rotation. The single-electron Hamiltonian for each spin flavor in valley + can be written as

$H_{+}=\sum_{\boldsymbol{k}} \left[ \psi_{\boldsymbol{k},+,t}^{\dagger}h_{-\theta/2}^{t}\left( \boldsymbol{k}-\boldsymbol{K}_{+}^{t} \right)\psi_{\boldsymbol{k},+,t}+\psi_{\boldsymbol{k},+,b}^{\dagger}h_{\theta/2}^{b}\left( \boldsymbol{k}-\boldsymbol{K}_{+}^{b} \right)\psi_{\boldsymbol{k},+,b}+\sum_{j=0}^{2} \left( {\psi_{\boldsymbol{k}-\boldsymbol{q}_{0}+\boldsymbol{q}_{j},+,t}^{\dagger}T}_{j}\psi_{\boldsymbol{k},+,b}+h.c. \right) \right]$,

where the momenta are measured from the center of the MBZ, $\psi_{\boldsymbol{k},+,t}^{\dagger}$ ($\psi_{\boldsymbol{k},+,b}^{\dagger}$) is the two-component (four-component) creation operator of electrons in the AB (ABAB) sublattice basis for the top (bottom) sheet, and $h_{\theta}^{t/b}\left( \boldsymbol{k} \right)\equiv h^{t/b}(R_{\theta}\boldsymbol{k})$. For the top sheet, we choose $h^{t}(\boldsymbol{k})$ as the $2\times2$ Dirac Hamiltonian of monolayer graphene. For the bottom sheet, we choose $h^{b}(\boldsymbol{k})$ as the $4\times4$ effective Hamiltonian of bilayer graphene, whose details can be found in Refs. [2,4]. The $\boldsymbol{q}$ vectors are $\boldsymbol{q}_{0}=\boldsymbol{K}_{+}^{b}-\boldsymbol{K}_{+}^{t}$, $\boldsymbol{q}_{1}=R_{2\pi/3}\boldsymbol{q}_{0}$, and $\boldsymbol{q}_{2}=R_{-2\pi/3}\boldsymbol{q}_{0}$ (Figure S1). The moiré tunneling between top and bottom sheets is given by $T_{j}=w_{0}+w_{1}e^{i(2\pi/3)j\sigma_{z}}\sigma_{x}e^{-i(2\pi/3)j\sigma_{z}}$, where $w_{0}$ and $w_{1}$ are the tunneling between the same and different sublattices, respectively, and $\sigma$’s are the Pauli matrices in the sublattice basis.

In our band structure calculations, we use the phenomenological parameters extracted in Ref. [4] for the graphene sheets. To take into account the lattice relaxation effect, we set $w_{1}=110 \mathrm{meV}$ and $w_{0}=0.6w_{1}$. For the three graphene layers from top to bottom, we add electrostatic potentials $(\frac{U}{2}, 0, -\frac{U}{2})$ to $H_{+}$ to capture the effect of a vertical displacement field $D$. We have $U=-eDd/\varepsilon$, where $\varepsilon\approx3.9$ and $d\approx1 \mathrm{nm}$ are the dielectric constant and thickness of our sample, respectively.


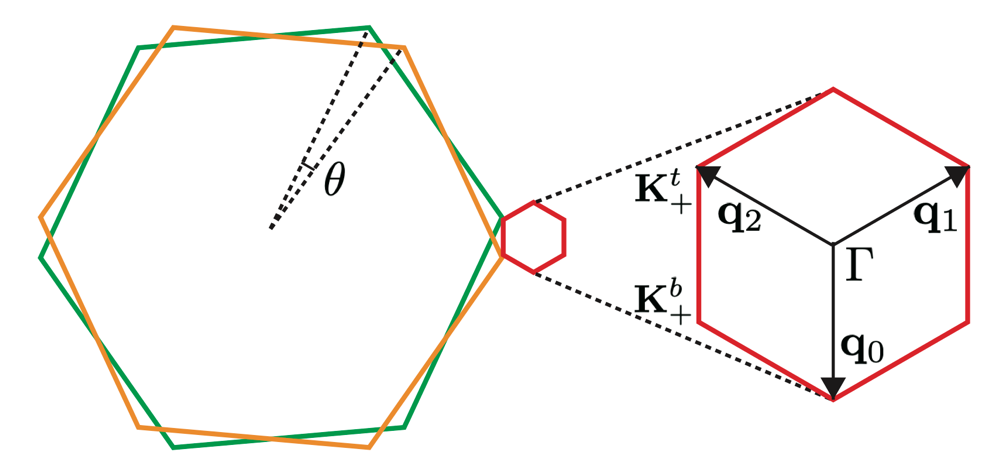


**Figure S1**

Moiré Brillouin zone of TMBG in valley +. The two large hexagons (green and orange) are original first Brillouin zones of monolayer graphene and bilayer graphene. The small red hexagon represents the MBZ in the valley + resulting from twist.

Note 2: Band structure of TMBG with a twist angle of 1.67°.

In the main text, the band structure of TMBG at a twist angle of 1.67° (Dev. 1) is depicted with and without a displacement field. Here, band diagrams of Dev. 1 under various displacement fields are calculated and further analyzed (Figure S2). These results consistently show the superlattice-induced bandgaps above and below the flat bands, alongside with an additional gap near the charge neutrality point which widens with the increasing displacement field magnitude. Notably, due to the disrupted inversion symmetry along the z-direction in TMBG, the band structures exhibit differences between positive and negative displacement fields.^[5]^ Specifically, a larger bandgap near the charge neutrality point is evident for negative displacement fields compared to positive ones of the same magnitude. This observation aligns with patterns seen in the transport measurement maps (Figure 1c in the main text), where resistance is higher under negative displacement fields at the charge neutrality point.


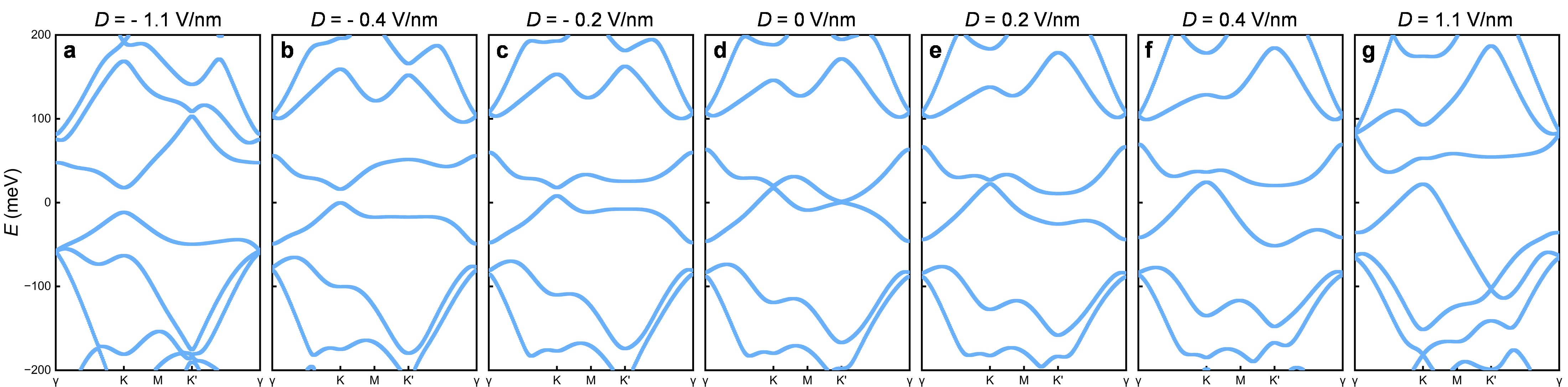


**Figure S2**

Band structure of TMBG (Dev. 1) under different displacement fields. a-g) Band structures of Dev. 1 with a twist angle of $\theta=1.67^{\circ}$ under varying displacement field conditions: $D=-1.1 V/nm$ (a), $D=-0.4 V/nm$ (b), $D=-0.2 V/nm$ (c), $D=0 V/nm$ (d), $D=0.2 V/nm$ (e), $D=0.4 V/nm$ (f) and $D=1.1 V/nm$ (g).

Experimentally, we conducted a temperature-dependent analysis of the two-probe electrical transport near the band gaps of Dev.1. Using the Arrhenius model (ln(*ρ*) = −*E*_g_/2*k*_B_*T* + *C*, where *ρ* is resistivity, *k*_B_ is the Boltzmann constant, *C* is a constant, and *E*_g_ is the electrical band gap), we plotted ln(*R*) versus 1/*T* and carried out linear fits to quantify the gaps. At the charge neutrality point under a displacement field of *D* = −1.1 V/nm, the derived gap was 11.4 meV. A similar analysis at the flat-band filling position (*D* = 0) yielded a gap of 27.2 meV. These experimental values are smaller than the corresponding theoretical predictions (29.4 meV and 39.6 meV, respectively). We attribute this discrepancy to residual contact resistance (*R*_contact_) in the two-probe configuration, where the total measured resistance (*R*) combines *R*_contact_ and the intrinsic sample resistance (*R*_sample_). Isolating *R*_contact_ and its temperature dependence is challenging; however, if assumed constant, *R*_contact_ would reduce the slope of the ln(*R*)–1/*T* curve, leading to an underestimated *E*_g_. While this analysis does not yield exact quantitative measurements, it still delivers strong qualitative evidence of bandgap formation at both the primary Dirac point and the superlattice gap.

**
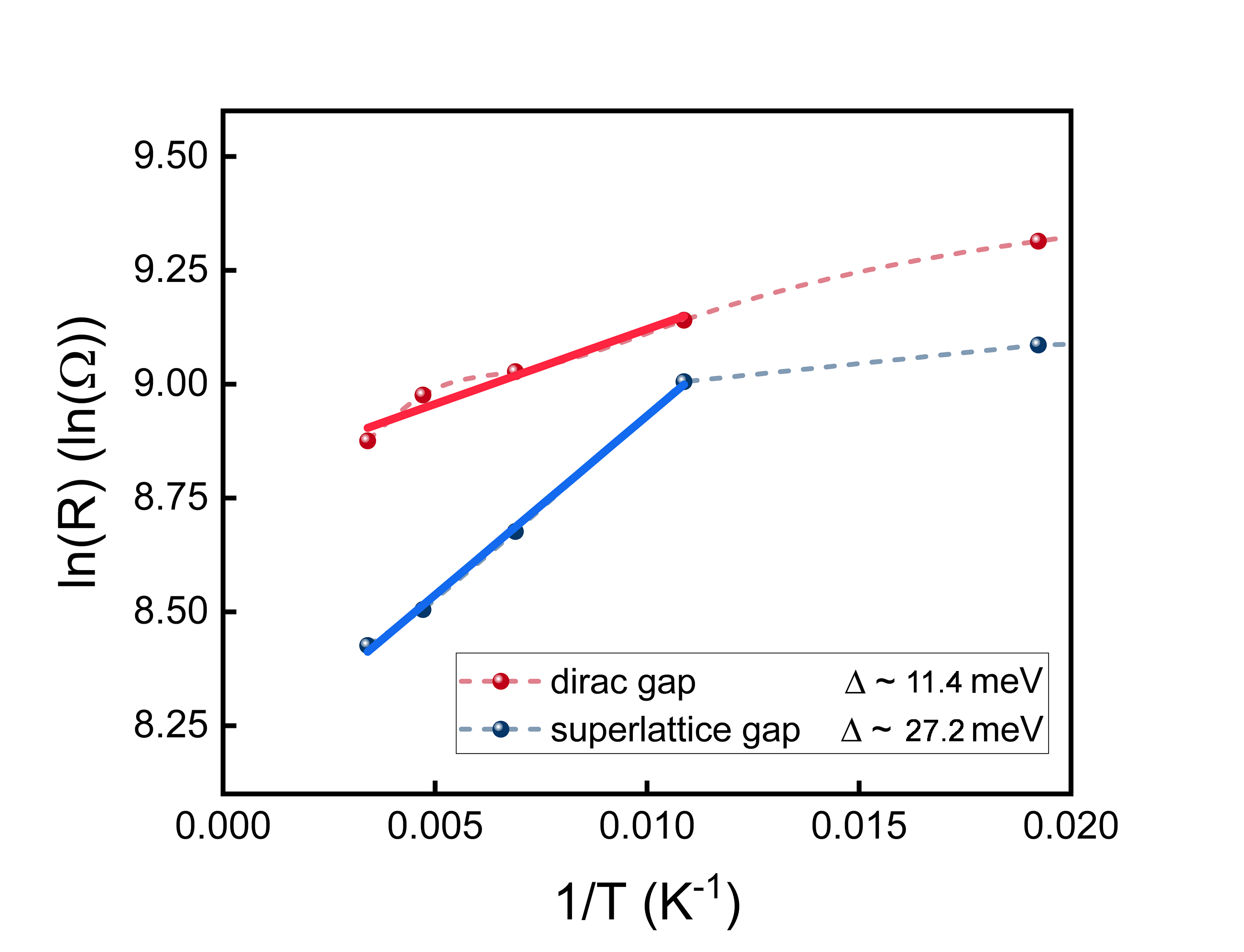
**

**Figure S3**

Temperature-dependent resistance analysis. Arrhenius plots showing ln(*R*) versus 1/*T* at the Dirac gap (red circles) and superlattice gap (blue circles). Dashed lines indicate visual guides, while solid red and blue lines represent linear fits to each dataset. Extracted gap values are shown in the inset.

Note 3: Twist angle dependence of the band structure.

We performed band structure calculations for TMBG with twist angles ranging from 1° to 5°, in order to investigate the evolution of moiré band properties with interlayer rotation. Our theoretical calculations reveal that within certain ranges (1° – 2.5°), smaller twist angles result in flatter moiré energy bands. These flat bands are significant because they can induce larger Seebeck coefficients and temperature coefficient of the conductance, which are pivotal for enhancing photothermoelectric and bolometric effects in photodetection. This behavior is detailed in Note 10, where we discuss the implications of these flat bands for device performance.

**Figure S4**

Calculated band structures of TMBG with varying small twist angles. a–d) Energy band diagrams of TMBG for twist angles of 1° (a), 1.67° (b), 2° (c), and 2.5° (d), respectively.

At even larger twist angles, the two graphene layers tend to decouple. This decoupling leads to the absence of flat bands and superlattice-induced band gaps, which in turn reduces the magnitude of thermal effects observable in devices with smaller angles. The absence of these flat bands means that the interlayer interactions are minimized, resulting in band structures that resemble those of isolated graphene sheets, thereby limiting the enhancement of photoresponse through interlayer electronic interactions.

**Figure S5**

Calculated band structures of TMBG with varying larger twist angles. a–c) Energy band diagrams of TMBG for twist angles of 3° (a), 4° (b), 5° (c), respectively.

Note 4: Determination of sub-terahertz incident power.

To determine the incident power of the sub-terahertz laser on the device, we first analyze the spatial distribution of light intensity using a terahertz camera (Swiss Terahertz; Rigi Camera) positioned at the device’s height to visualize the light spot shape. The laser beam is directed onto the camera via an off-axis parabolic mirror, revealing a Gaussian distribution of light intensity described by the functional form: $f=ae^{-\frac{r^{2}}{2c^{2}}}$. Afterwards, the camera is replaced with a power meter (Ophir; RM9-THz) featuring a tunable diaphragm diameter to measure beam power across various settings. Table S1 shows the results taken on Dev. 2. The data is utilized to fit the Gaussian function, thereby determining parameters *a* and *c*.

**Table S1: Diaphragm diameter and the corresponding measured beam power**

| diaphragm diameter (mm) | 3 | 4 | 5 | 6 | 7 | 8 |
| --- | --- | --- | --- | --- | --- | --- |
| beam power (mW) | 7 | 8.75 | 10.11 | 10.51 | 10.8 | 11.2 |

To determine the laser power incident on the device, we define the active area as the graphene region between the source (S) and drain (D) electrodes. In the case of Dev. 2, this active area forms a rectangle measuring 7 μm × 6 μm. To simplify the calculation of the incident power density, we approximate this rectangular area as a circle with an equivalent surface area of 42 μm², yielding an effective radius of ~3.66 μm. This approximation is justified because the active area is significantly smaller than the sub-terahertz laser’s spot size (radius ≈ 6000 μm). As a result, the error in estimating the power distribution is negligible.

Substituting this radius into the equation $P=2\pi ac^{2}(1-e^{-\frac{r^{2}}{2c^{2}}})$, the calculated incident powers for Dev. 1 and Dev. 2 are $31.6 \mathrm{nW}$ and $62.7 \mathrm{nW}$, respectively. This results in average power density of $743 {\mu W}/{\mathrm{mm}^{2}}$ and $1.49 mW/\mathrm{mm}^{2}$.

It should be noted that several uncertainties affect the determination of laser power. For instance, discrepancy may arise between the actual height of the sample and where the power meter was positioned. Namely, achieving precise placement of the device at the focal plane of the off-axis parabolic mirror is challenging, potentially leading to lower incident power on the device than expected. Additionally, positioning the sample precisely at the center of the laser spot is difficult; despite using a piezo positioning controller (Attocube; ANC300), manual displacement limitations and small sample size may prevent optimal alignment at the peak power density location. These factors tend to overestimate the incident power relative to the actual value, suggesting that the reported device responsivity could potentially exceed the values documented in the manuscript.

Another important consideration is the potential antenna effect of the metal electrodes, which could amplify the sub-THz electric field distribution within the graphene region. To investigate this, we conducted simulations of the electromagnetic field distribution at 0.3 THz. As illustrated in Figure S6, our experimental electrode geometry shows no significant field enhancement in the graphene channel. In comparison, a bowtie antenna (Figure S6b) specifically designed for 0.3 THz demonstrates a substantial enhancement (~100×). These results confirm that the as-fabricated contacts in our device do not act as efficient antennas.

Additionally, since antenna effects are highly polarization-sensitive, we simulated the bowtie structure under orthogonal (y-direction) polarization (Figure S6c), which revealed no enhancement. In our experimental setup, there was no intentional alignment of the THz beam’s polarization with the device. Despite this, all measured TMBG devices with similar graphene areas exhibited consistent photocurrent levels, further validating the absence of antenna-enhancing effects in our device’s geometry.

**Figure S6**

Simulation of the electric field distribution at sub-terahertz frequency (f = 0.3 THz) excitation. a) The electric field distribution at sub-terahertz frequency excitation for the device’s geometry used in the experiment. b,c) The electric field distribution for a typical bowtie antenna and graphene under x-polarized (b) and y-polarized (c) terahertz illumination. The white dashed lines mark the graphene area, and the z-axis represents the normalized electric field.

Note 5: Mid-infrared photoresponse of Dev. 3.

To characterize the photoresponse of our TMBG device in the mid-infrared (MIR) range, we employed a 10.6 µm wavelength MIR laser and developed a mid-infrared confocal microscopy platform equipped with a reflective objective lens. Another TMBG device with a 1.7° twist angle (Dev. 3) was fabricated to measure the photocurrent under MIR excitation, and the results were compared with its response under near-infrared excitation at the wavelength of 912 nm. The experimental findings are presented in Figure S7. Figure S7a displays the device’s resistance as a function of carrier density *n* and displacement field *D*, consistent with the behavior observed in Dev. 1 as discussed in the main text. Figures S7b to S7e illustrate the MIR and NIR photoresponse as a function of *D* and *n* under both zero bias and non-zero bias conditions. The MIR photocurrent maps exhibited characteristics similar to those observed under the 912 nm NIR laser, in both zero and non-zero bias scenarios, demonstrating that the TMBG transistor also possesses photodetection capabilities in the mid-infrared range. The underlying photodetection mechanism remains consistent with that in the near-infrared range: the PTE effect under zero bias and the bolometric effect under applied bias.

**Figure S7**

Mid-infrared and near-infrared photocurrent spectra of Dev. 3. a) Two-probe resistance (*R*) map of Dev.3 as a function of *n* and *D* at $T=4.5 K$. b,c) Photocurrent maps as a function of *n* and *D* at $V_{\mathrm{DS}}=0$, under illumination at wavelengths of 10.6 μm with a power of 1.1 mW (b) and 912 nm with a power of 10 μW (c). d,e) Differential photocurrent maps as a function of *n* and *D* obtained by subtracting the response at zero bias from that at $V_{\mathrm{DS}}=30 \mathrm{mV}$, for the corresponding wavelengths of 10.6 μm (d) and 912 nm (e).

Mid-infrared SPCM measurement was also performed on Dev. 3. Since the MIR laser spot is much larger than the device’s active area, the entire TMBG area collectively contributes to the photocurrent under excitation, with the net signal arising from the integration of responses across asymmetric regions. Figure S8 presents the SPCM image of the device (zero-bias configuration) with a ~ $5 \times5 {\mu m}^{2}$ photosensitive area. The observed photocurrent map reveals a single bright spot corresponding to the laser beam profile. The photoresponse is strongest when the device is centrally aligned with the MIR spot while gradually diminishes as the device shifts toward the periphery of the beam. This spatially resolved photocurrent profile confirms the intrinsic asymmetry-driven photoresponse of the TMBG device, validating its suitability for terahertz detection under uniform illumination.

**Figure S8**

Spatial mid-infrared photocurrent map of Dev. 3. The photocurrent map was obtained using a 10.6 µm laser, with the following bias and gate voltage configuration: $V_{\mathrm{DS}} = 0 mV$, $V_{\mathrm{TG}} = -4.5 V$, and $V_{\mathrm{BG}} = -2.6 V$. The measurement was conducted at a temperature of 4.5 K.

Note 6: Locally excited photothermoelectric response in Dev. 1.

In the main text, the zero bias photocurrent in Dev. 1 is attributed to the photothermoelectric (PTE) effect, arising from the diffusion of photo-excited hot carriers along a temperature gradient. Consequently, the photocurrent polarity reverses as the light spot moves across the device. Figure S9a displays the zero bias photocurrent of Dev. 1 under a near infrared laser focused on the source electrode side. Two 'S' shaped patterns in the photocurrent (marked with grey dashed lines) are observed as the back gate voltage varies, reflecting changes in the Seebeck coefficient near the bandgap regions. Similarly, when the beam spot shifts to the drain electrode side (Figure S9b), a mirrored photocurrent curve with opposite polarity is obtained, consistent with the PTE mechanism.^[6]^ It's noted that the photocurrent magnitudes differ between Figure S9a and S9b, possibly due to asymmetries introduced during device fabrication. This asymmetry results in a net PTE signal under uniform device illumination.


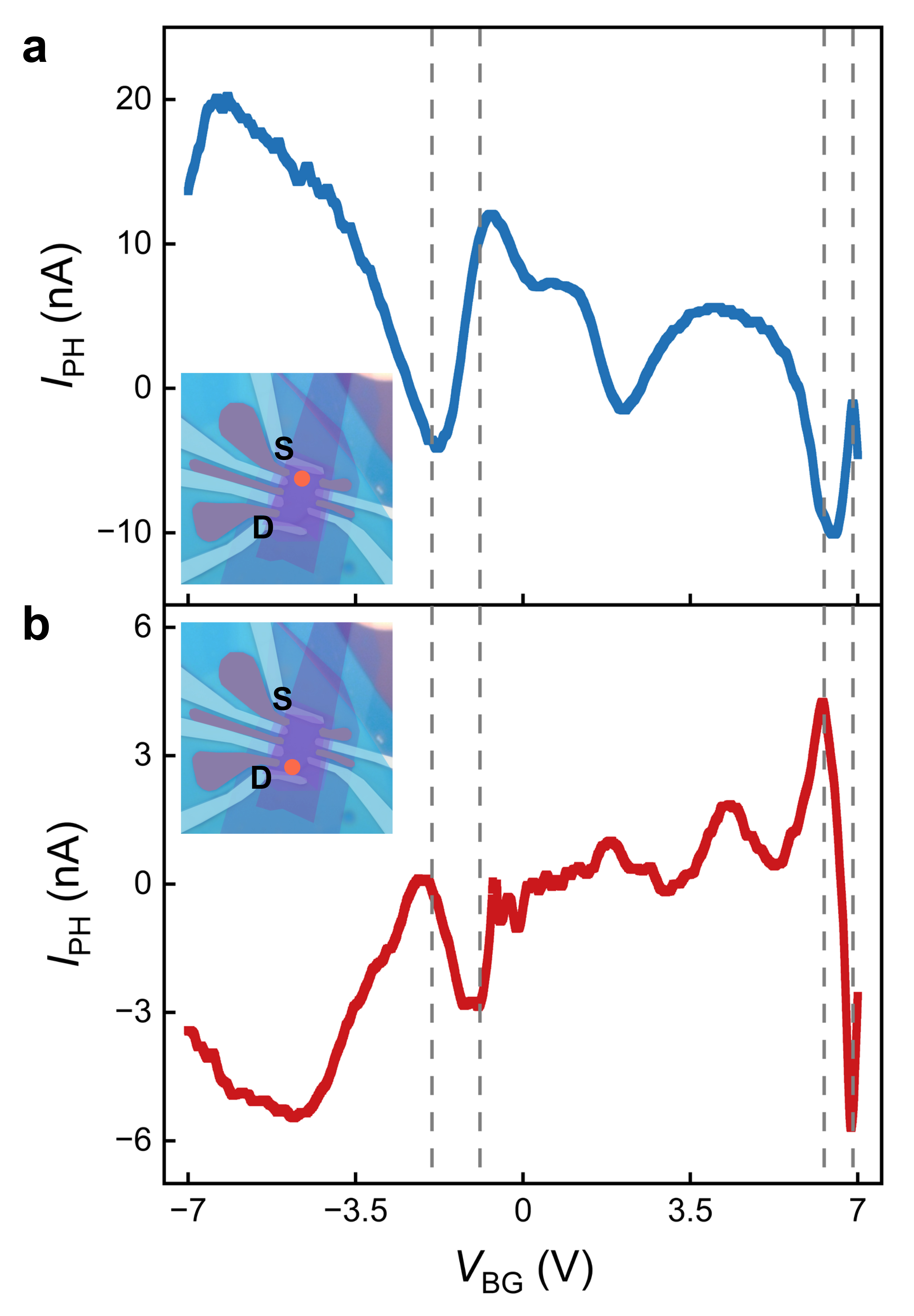


**Figure S9**

Locally excited photothermoelectric response of Dev. 1. a-b) Photocurrent as a function of *V*_BG_, with the light spot focused near the source electrode (a) and drain electrode (b), respectively. The measurement employed a light source operating at a wavelength of $912 \mathrm{nm}$ and a power of $12 \mu W$, conducted at a temperature of $4.5 K$ with the top gate voltage set to $0 V$.

To further validate the PTE mechanism, we analyzed whether the polarity of the photocurrent predicted by the PTE model is consistent with the experimental data (Figure S9a). In a one-dimensional conductive channel, the PTE-induced photocurrent can be described by:

$$I_{\mathrm{PTE}}=-\frac{1}{R}\int S\left( x \right)\nabla T_{e}\left( x \right)dx$$

where $S(x)$ is the spatially varying Seebeck coefficient, $T_{e}(x)$ is the local electron temperature, and *R* is the device resistance.

As illustrated in Figure S10b, localized electron heating under illumination generates a temperature gradient (∇*T*) within the device. The metallic electrode acts as a thermal sink, maintaining a base temperature and the temperature drops sharply near the illuminated contact (with relatively large temperature gradient as depicted in Figure S10c). The Seebeck coefficient profile is influenced by doping heterogeneity and can be qualitatively described using the following simplified model. Near the Au electrode (work function: 5.2 eV), graphene is heavily p-doped (*p*+) due to Fermi-level pinning, yielding a gate-voltage-independent positive Seebeck coefficient (*S*_1_). This *p*+-doped region extends at least 100 nm into the channel (Figure S10d).^[7]^ Beyond this zone, the Seebeck coefficient (*S*_2_) becomes gate-tunable and uniform across the central part of the channel. The expression for the photocurrent then simplifies to:

$$I_{\mathrm{PTE}} \sim\left( S_{2}-S_{1} \right)\nabla T_{x2}+\left( S_{1}-S_{2} \right)\nabla T_{x3}-S_{1}(\nabla T_{x4}-\nabla T_{x1})$$

Here, $\nabla T_{\mathrm{xi}}$ is the temperature gradient in the respective regions shown in Figure S10. In this configuration, the sign of the photocurrent is determined by the relative magnitudes of *S*_2_ and *S*_1_ as well as the direction of the temperature gradient.

Experimentally, at *V*_BG_ = -2 V and *V*_TG_ = 0 V (lightly p-doped channel), the photocurrent is negative (Figure S9a). This aligns with our model: when the channel’s *S*_2_ > *S*_1_ and illumination is applied near the source electrode, the integrated *S*(*x*)⋅∇*T*(*x*) yields a negative response. Conversely, under *n*-doped conditions (*V*_BG_ = -0.8 V and *V*_TG_ = 0 V), the reversal of *S*_2_ polarity produces a positive photocurrent, consistent with the experimentally-observed photoresponse peak. This agreement between theory and experiment confirms that the photocurrent sign is governed by the interplay of doping-dependent Seebeck coefficients and illumination-induced thermal gradients, validating our PTE-dominated mechanism.

**Figure S10**

Analysis of the photothermoelectric current’s sign. a) Schematic of the device structure showing source (S) and drain (D) configurations with a red dot indicating the position of the light spot as simulated in Figure S9a of the Supplementary Information. b) Temperature distribution along the X-axis showing a sharp temperature gradient in the vicinity of the S electrode and a gentler gradient extending toward the D electrode. c) Temperature gradient (∇T) across the device, with a more pronounced gradient near the S electrode. d) The Seebeck coefficient (S) varies along the X-axis when the device is modulated to p-type doping by the gate voltage.

To further validate that the photoresponse under sub-THz illumination originates from the PTE effect, and the PTE response of TMBG devices under uniform illumination arises from the collective contribution of individual regions near the graphene-metal contacts, we compared the sub-THz photocurrent in Dev. 4 (a dual-gated TMBG transistor with a 1.6° twist angle, see inset of Figure S11a) before and after selectively shadowing one graphene-metal contact.

Under uniform sub-THz irradiation, the zero-bias photocurrent exhibits an ‘S’-shaped dependence on bottom-gate voltage near the charge-neutrality point (Figure S11a) and the superlattice-induced band gap (Figure S11b), consistent with the gate-tunable Seebeck coefficient of TMBG. This response reflects the summation of opposing-polarity currents generated asymmetrically at the two contacts. To isolate contributions from individual contacts, we shadowed the contact with stronger intrinsic photoresponse by depositing a 2 × 3 µm Au mask over it (inset of Figure S11c). Post-shadowing, the photocurrent near both the charge-neutrality point and superlattice bandgap reversed its gate dependence (orange curves, Figure S11c-d), transitioning from a high-to-low ‘S’ shape as the bottom-gate voltage increased. The reversed trend confirms that shadowing a single contact suppresses its local photothermoelectric contribution, directly linking the net photocurrent to the asymmetric interplay between contacts. While the additional fabrication steps (electron-beam lithography and Au deposition) likely degraded device quality—preventing significant responsivity enhancement—the inversion of the gate-dependent response robustly validates our model of spatially resolved photocurrent summation.

These results demonstrate that the sub-THz photoresponse originates from the collective, asymmetric contributions of the graphene-metal contact regions. Optimizing device geometry to enhance this asymmetry could, in principle, improve responsivity of the device.

**Figure S11**

Sub-terahertz photoresponse before and after electrode masking. a,b) Photocurrent generated by the unshadowed device as a function of *V*_BG_ at a fixed top gate voltage of $V_{\mathrm{TG}}=3 V$ (a) and $V_{\mathrm{TG}}=1 V$ (b), respectively. c,d) Photocurrent generated by the device with one electrode shadowed, as a function of *V*_BG_ at the same fixed $V_{\mathrm{TG}}=3 V$ (c) and $V_{\mathrm{TG}}=1 V$ (d), respectively. Optical microscope images of the unshadowed (a, inset) and shadowed (c, inset) device are shown for reference.

Note 7: Temperature dependent transport properties of Dev.1.

The transport properties of Dev. 1 at elevated temperatures are characterized in Figure S12. Resistance of the device is plotted against both back gate and top gate voltages at $T=32 K$ (Figure S12a) and $92 K$ (Figure S12b). With increasing temperature, the peak resistance decreases as the Fermi level aligns within the bandgap, and the resistance map shows broader peaks due to the thermal expansion of the band. Notably, the resistance peaks associated with superlattice-induced bandgaps persist up to $92 K$, as depicted in Figure S12b. It is worth mentioning that the calculated $TCC$ at $4.5 K$ in the main text (Figure 3d) is determined from the transport data at $32 K$ (Figure S12a) and $4.5 K$ (Figure 1c), using the formula $\frac{1}{G}\frac{\Delta G}{\Delta T}$.


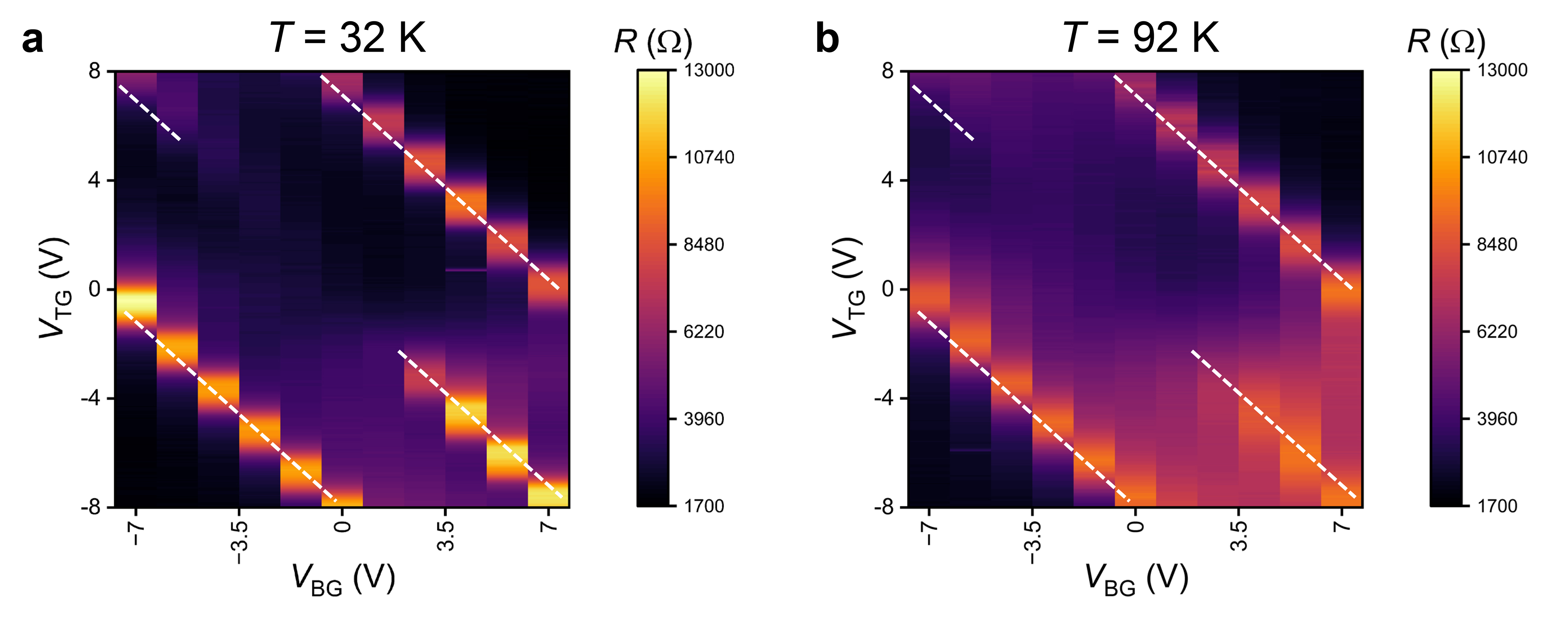


**Figure S12**

Transport characterizations of Dev. 1 at elevated temperatures. a-b) Two-probe resistance maps of Dev. 1 as a function of *V*_TG_ and *V*_BG_ at temperatures of $32 K$ (a) and $92 K$ (b). The white dashed lines, representing high-resistance states observed in the transport characteristics at $4.5 K$ (Figure 1c), aid in temperature-based comparisons.

Note 8: Additional characterization of the bolometric response in Dev. 1.

In the main text, the bolometric photoresponse of Dev. 1 is characterized by generating differential photocurrent maps (Figure 2d-2f), obtained by subtracting the photocurrent at a DC bias of $30 \mathrm{mV}$ from that without bias. Here, we present the differential photocurrent map as a function of top and back gate voltages under near-infrared laser excitation at $-30 \mathrm{mV}$ bias, subtracting the zero bias map using the same methodology as Figure 2d-f. Compared to Figure 2e, the overall polarity of the photoresponse map reverses, while its characteristics remain consistent. Notably, significant photocurrent with opposite polarity occurs as the Fermi level shifts into and out of the bandgaps. To further investigate bias impact on the photoresponse, we select various gate configurations and measure photocurrent as a function of the bias voltage, as illustrated in Figure S13b. The curves show a negative slope when the Fermi level is within the flat moiré band (top left panel) or conduction band (bottom left panel), and a positive slope when within the bandgap near the charge neutrality point (top right panel) or superlattice-induced bandgap (bottom right panel). This behavior is consistent with the photo-bolometric mechanism, where the slope of the curves represents $\Delta G$ (where $\Delta G=G\cdot TCC\cdot\Delta T$), showing opposite temperature dependencies between metallic (left panels) and insulating (right panels) conditions of the TMBG.


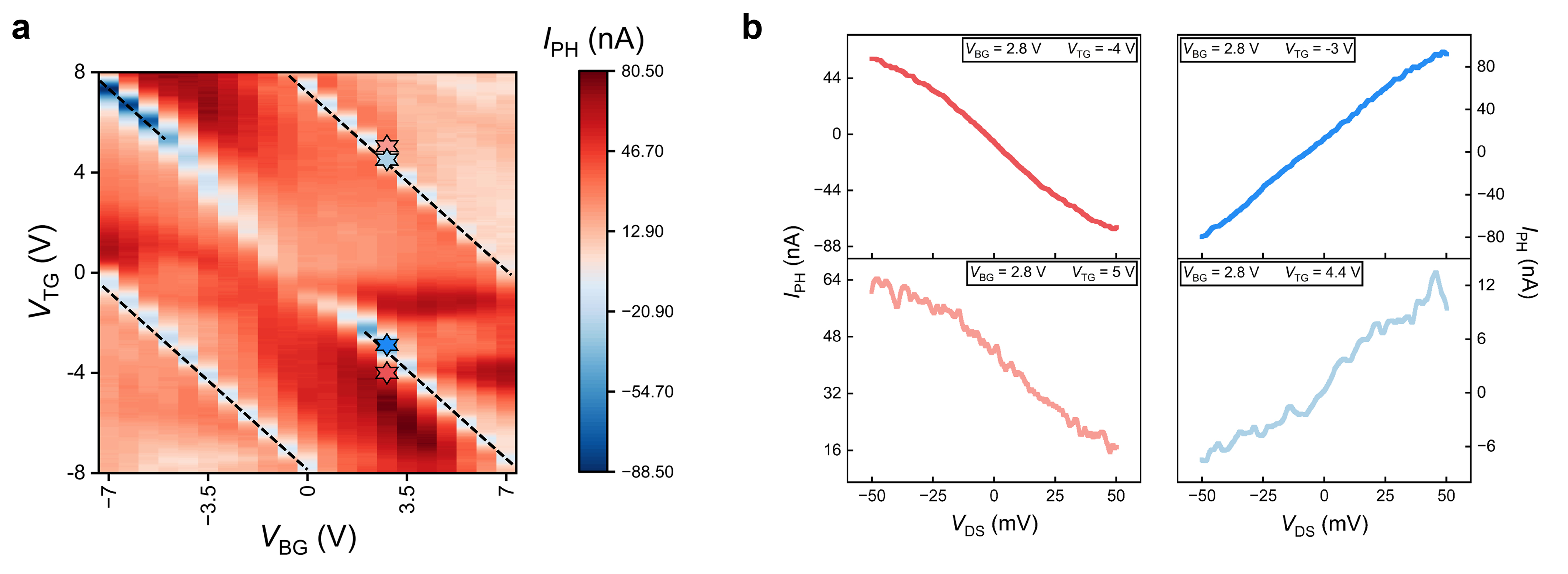


**Figure S13**

Bias voltage dependent photocurrent of Dev. 1. a) Differential photocurrent map as a function of *V*_TG_ and *V*_BG_, obtained by subtracting the response at zero bias from that at a $-30 \mathrm{mV}$ bias (${(V}_{\mathrm{DS}}=-30 mV)-{(V}_{\mathrm{DS}}=0)$) under irradiation from a $912 \mathrm{nm}$ laser beam. b) Photocurrent as a function of *V*_DS_ at a fixed $V_{\mathrm{BG}}=2.8 V$ for selected top gate voltages: $V_{\mathrm{TG}}=-4 V$ (top left panel), $V_{\mathrm{TG}}=5 V$ (bottom left panel), $V_{\mathrm{TG}}=-3 V$ (top right panel), and $V_{\mathrm{TG}}=4.4 V$ (bottom right panel). Gate voltage configurations are marked with hexagram in corresponding colors in (a).

Note 9: Comparison between the photothermoelectric and bolometric response in Dev. 1.

To further identify the photoresponse mechanism under varying bias conditions, we employ scanning photocurrent microscopy (SPCM) for spatial visualization of the photocurrent distribution. The laser is scanned across the device using a customized two-axis piezo nanopositioner (Nano Motions Technology Co., Ltd.), with a spatial resolution of approximately $0.1 \mu m$. A step size of $0.5 \mu m$ is employed to capture photocurrent at various locations, generating a spatial map. Simultaneously, reflected light is detected by a silicon photodiode, converted to an electrical signal, and recorded by a lock-in amplifier synchronized with the chopper frequency, providing a reflection image of the device geometry.

Representative SPCM images are shown in Figure S14, with the Fermi level of TMBG within the superlattice-induced bandgap under different bias voltages. At zero bias (Figure S14a), the photoresponse with opposite polarity is observed near the source and drain electrodes, consistent with the photothermoelectric effect.^[8]^ As the bias is applied (Figure S14), the photoresponse is generated predominantly in the graphene region, with the peak at the sample center, consistent with the expected behavior from the bolometric effect.^[9]^


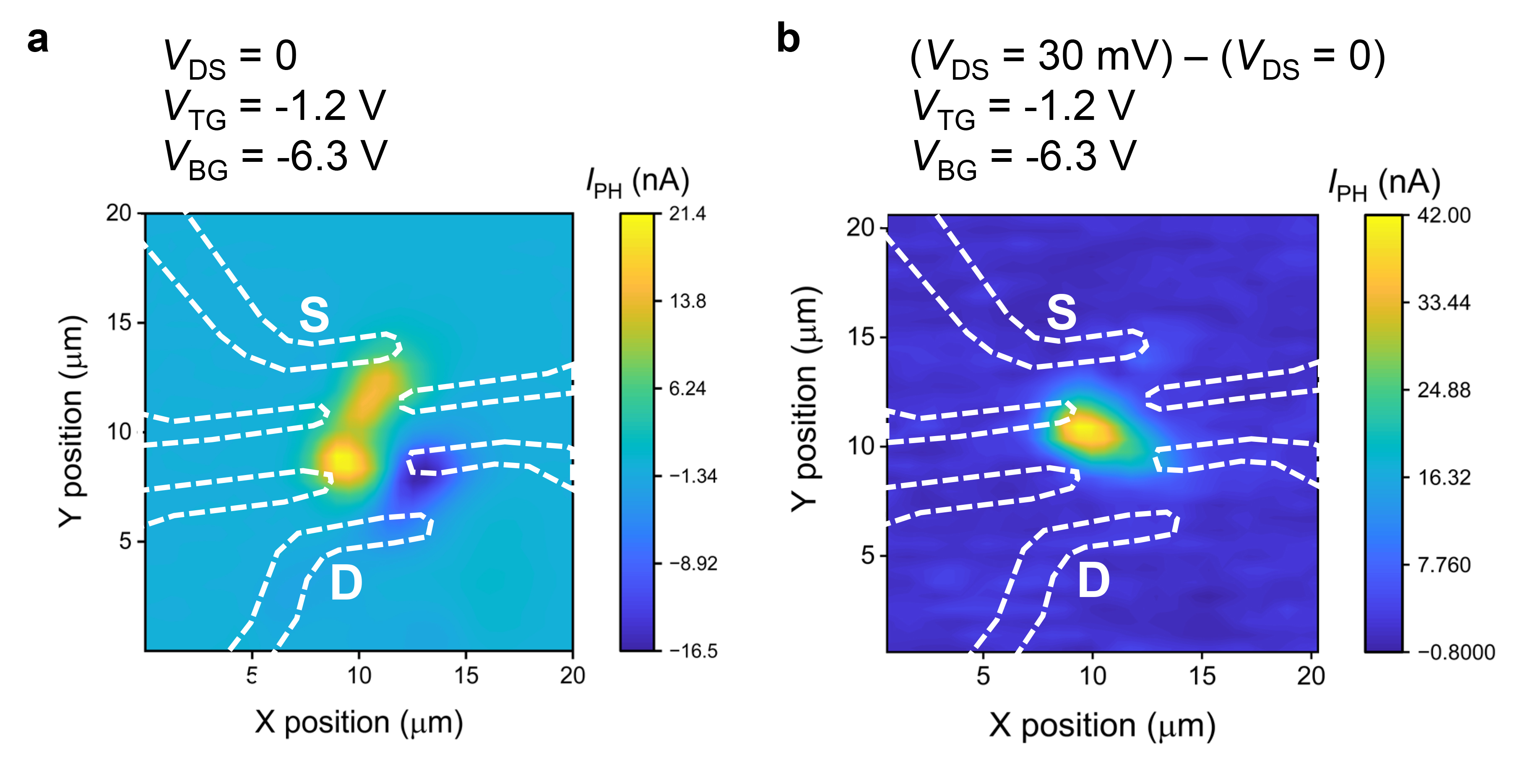


**Figure S14**

Spatial photocurrent maps of Dev.1. a) SPCM obtained at $V_{\mathrm{DS}}=0$, with $V_{\mathrm{TG}}=-1.2 V$ and $V_{\mathrm{BG}}=6.3 V$, using a near infrared laser as excitation. The electrodes are marked with white dashed lines. b) Differential SPCM obtained by subtracting the response at $0$ bias from that at a $30 \mathrm{mV}$ bias ($(V_{\mathrm{DS}}= 30 mV)-(V_{\mathrm{DS}}=0)$), with the other experimental parameters set the same as in (a). All measurements are carried out at a temperature of $4.5 K$.

Note 10: Temperature dependence of the sub-terahertz photoresponse in Dev.1.

To investigate the performance of the TMBG photodetector in a wide temperature range, the sub-terahertz photocurrent of Dev. 1 is measured across temperatures ranging from $4 K$ to $293 K$ at various bias and gate voltages (Figure S15). In general, the photocurrent decreases with increasing temperature. Specifically, at zero bias, the photocurrent smoothly decays below $150 K$ (Figure S15a). In contrast, the bias-voltage induced photocurrent exhibits more abrupt changes (Figure S15b), suggesting that the photocurrent due to the photothermoelectric effect is more resilient to temperature variations compared to that driven by the bolometric effect at low temperatures. Above $150 K$, the bolometric effect continues to decrease with temperature, while the photothermoelectric response tends to towards temperature independence.


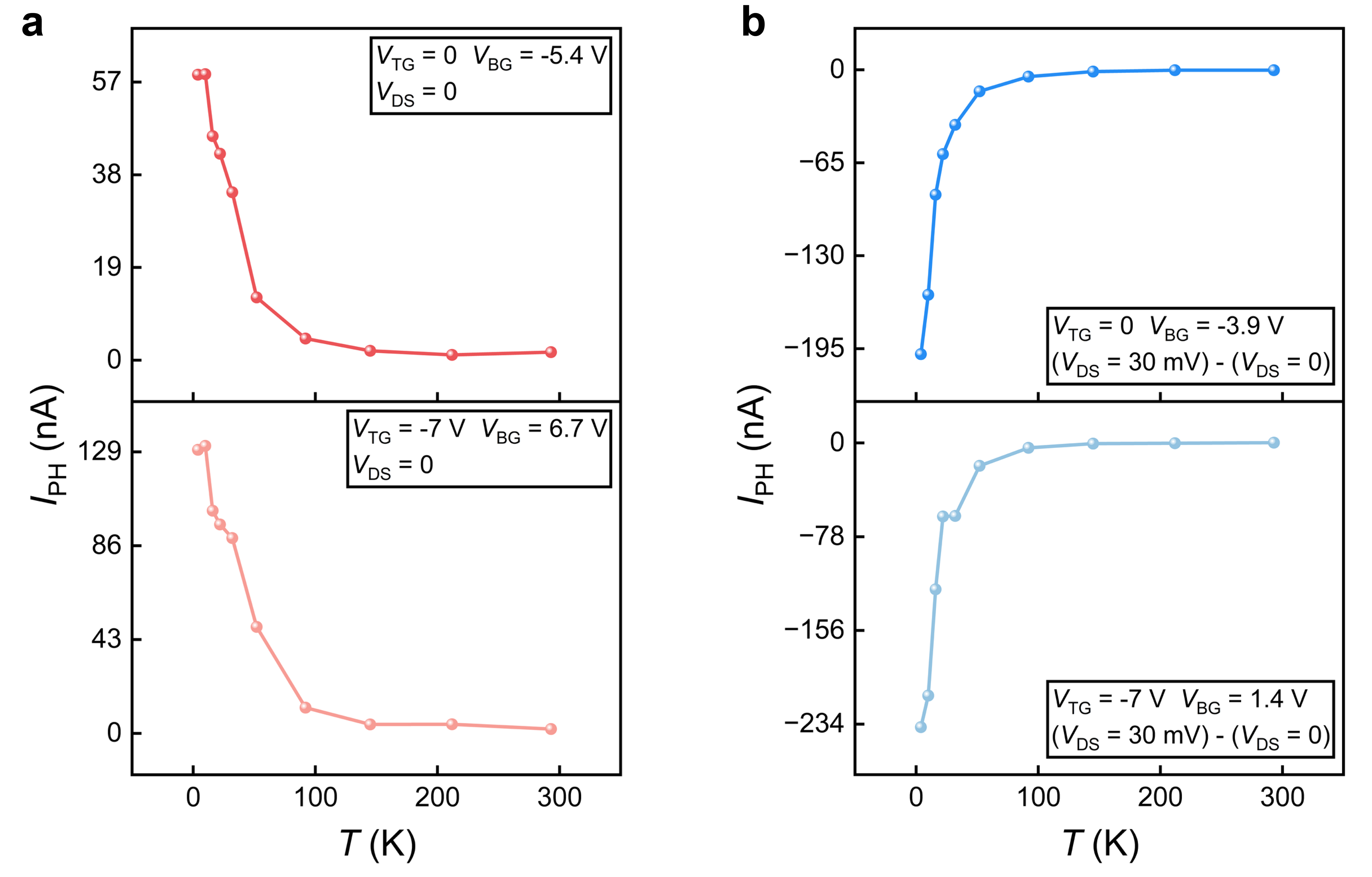


**Figure S15**

Temperature dependence of sub-terahertz photocurrent in Dev.1. a) Sub-terahertz photocurrent as a function of temperature (*T*) at $V_{\mathrm{DS}}=0$ with the gate configurations of$V_{\mathrm{BG}}=0$ and $V_{\mathrm{TG}}=-5.4 V$ (top panel); $V_{\mathrm{BG}}=-7 V$ and $V_{\mathrm{TG}}=6.7 V$ (bottom panel). b) Differential photocurrent measured by subtracting the response at $0$ bias from that at a $30 \mathrm{mV}$ bias ($(V_{\mathrm{DS}}=30 mV)-(V_{\mathrm{DS}}=0)$). The applied gate voltages are $V_{\mathrm{BG}}=0$ and $V_{\mathrm{TG}}=-3.9 V$ (top panel), $V_{\mathrm{BG}}=-7 V$ and $V_{\mathrm{TG}}=1.4 V$ (bottom panel), respectively. All the responses are excited using a sub-terahertz laser source at $0.3 \mathrm{THz}$.

The sub-terahertz photocurrent maps (as a function of the top and bottom gate voltages) are obtained with zero applied bias at temperatures of $32 K$ (Figure S16a) and $92 K$ (Figure S16b). Comparing with Figure 2c in the main text, it is evident that the photocurrent generally decreases as temperature increases, yet polarity inversions of the signal are still observed near the band gap. The term of the Seebeck coefficient ($\frac{1}{R}\frac{dR}{dV_{G}}$) is calculated at $32 K$ (Figure S16c) and $92 K$ (Figure S16d) using transport characteristics, as shown in Figure S16a and Figure S16b. The consistency between the observed photoresponse and the calculated $\frac{1}{R}\frac{dR}{dV_{G}}$ in Figure S16 confirms that the photothermoelectric effect sustains as the primary photoresponse mechanism in zero biased device up to a temperature of $92 K$.


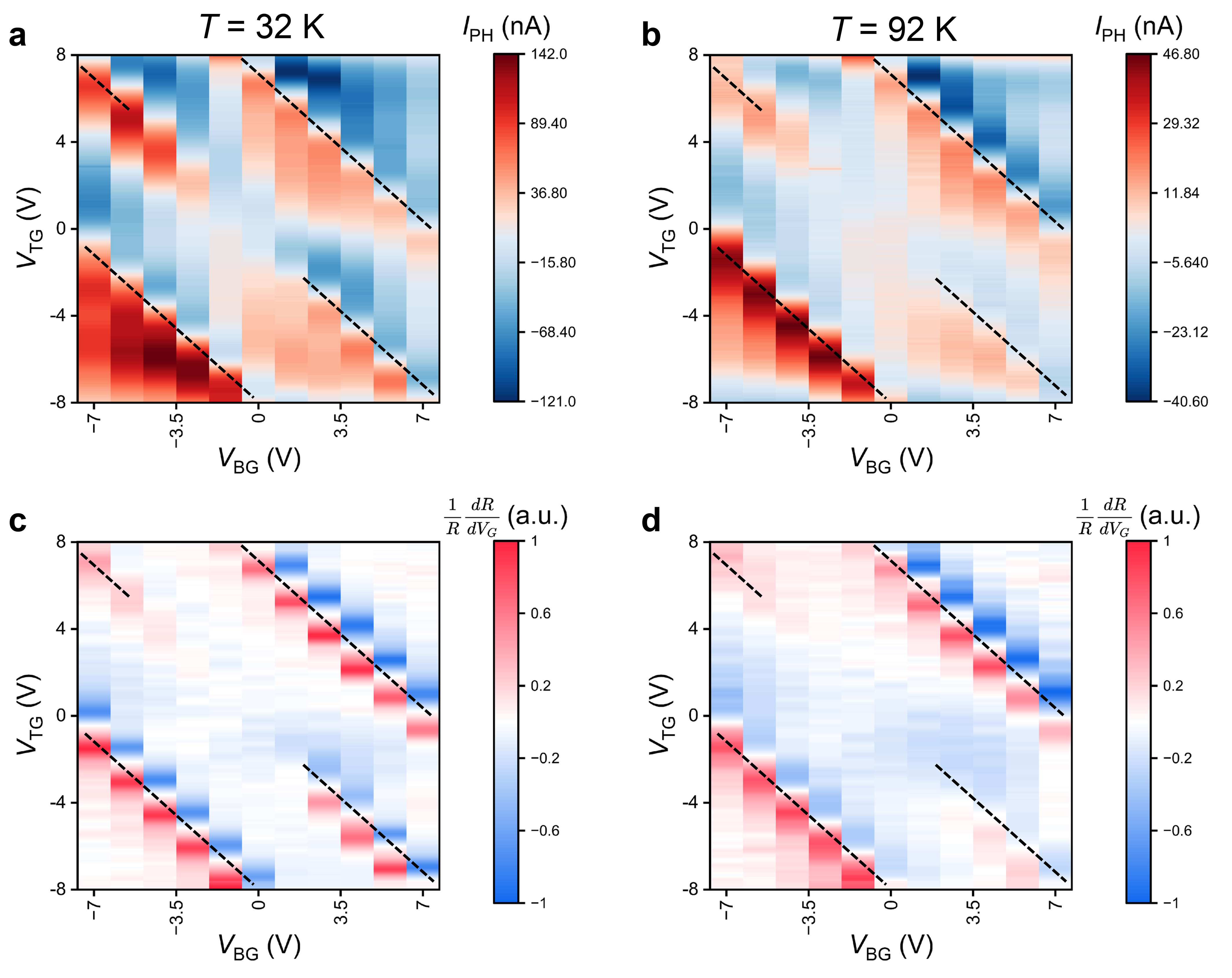


**Figure S16**

Photoresponse mechanism without bias at higher temperatures. a-b) Photocurrent maps as a function of *V*_TG_ and *V*_BG_ at $V_{\mathrm{DS}}=0$ with $T=32 K$ (a) and $T=92 K$ (b), under irradiation from a $0.3 \mathrm{THz}$ laser source. c-d) The term of Seebeck coefficient ($\frac{1}{R}\frac{dR}{dV_{G}}$) calculated as a function of *V*_TG_ and *V*_BG_ at $32 K$ (c) and$92 K$ (d), respectively.

Figure S17a illustrates the differential photocurrent map at $32 K$, obtained by subtracting the response at zero bias from that at $V_{\mathrm{DS}}=30 \mathrm{mV}$, as a function of *V*_TG_ and *V*_BG_. This map mirrors the earlier observations at lower temperatures, exhibiting a pronounced negative response within the flat moiré bands and a positive photocurrent within the bandgaps. Figure S17b presents the $TCC$calculated from the transport data at $32 K$ and $92 K$. The striking resemblance between Figure S17b and S17a highlights that, even at elevated temperatures, the bolometric effect continues to predominate as the photoresponse mechanism in the biased device.


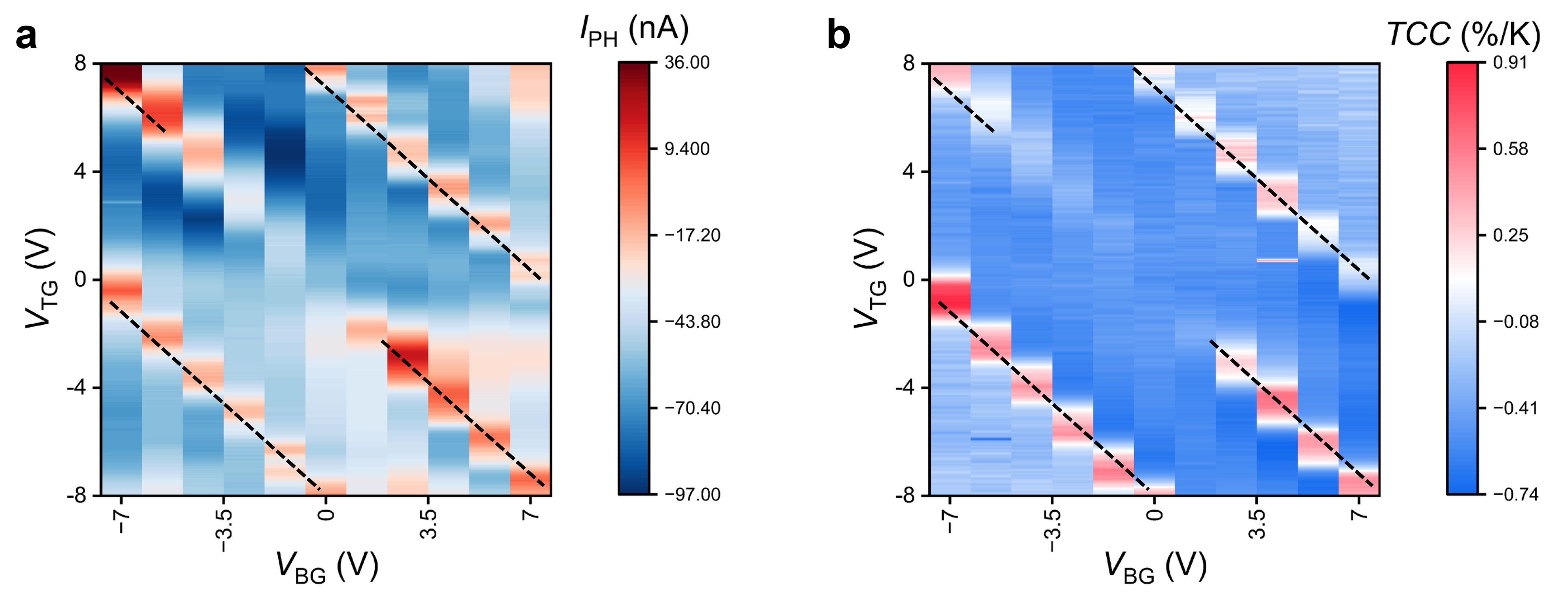


**Figure S17**

Photoresponse mechanism in biased device at higher temperature. a) Differential photocurrent map at $32 K$ obtained by subtracting the response at zero bias from that at a $30 \mathrm{mV}$ bias voltage (${(V}_{\mathrm{DS}}=30 mV)-{(V}_{\mathrm{DS}}=0)$) under irradiation from a $0.3 \mathrm{THz}$ laser source. b) The $TCC$ calculated as a function of *V*_TG_ and *V*_BG_ at $32 K$.

Note 11: Band structure of TMBG with a twist angle of 1.36°.

The band diagrams for Dev. 2 with a twist angle of 1.36° are depicted at different displacement fields in Figure S18. In contrast to the band structures of Dev. 1 (Figure 1d and Figure S2), the moiré bands appear notably flatter here due to the reduced twist angle. We observe superlattice-induced bandgaps above and below the flat bands, along with another distinct gap near the charge neutrality point that widens with increasing displacement field magnitude. These findings are consistent with previous calculations performed on Dev. 1.


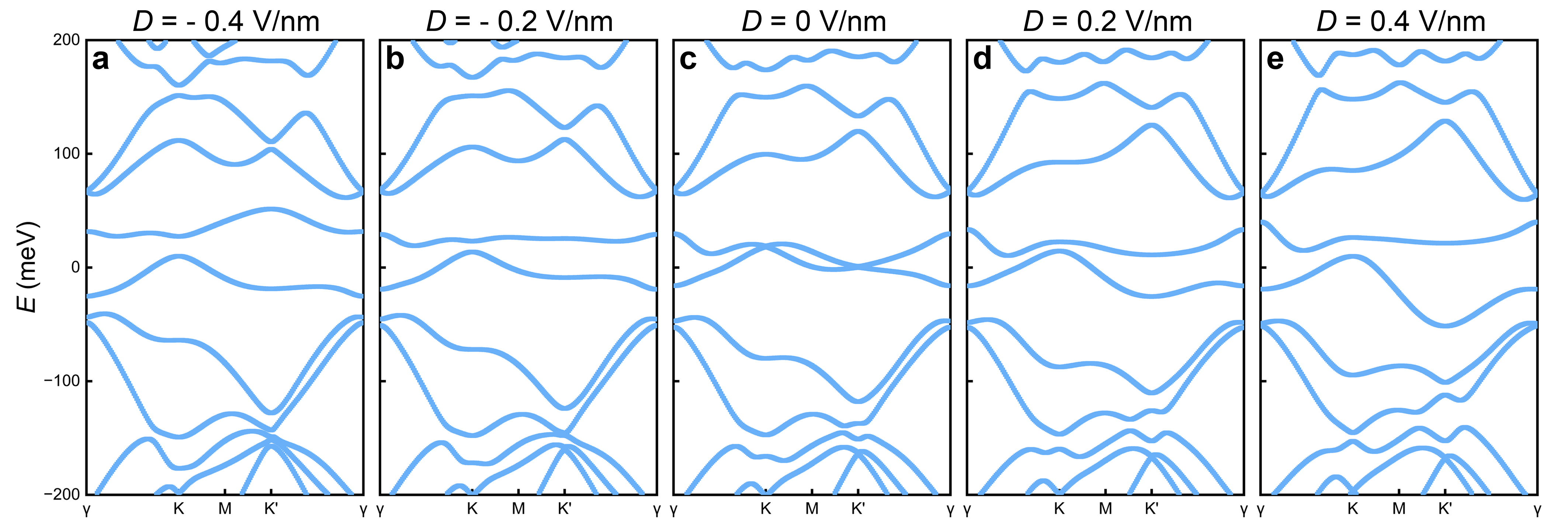


**Figure S18**

Band structures of TMBG (Dev. 2). a-e) The band structures of Dev. 2 with a twist angle of $\theta=1.36^{\circ}$ under varying displacement field conditions: $D=-0.4 V/nm$ (a), $D=-0.2 V/nm$ (b), $D=0$ (c), $D=0.2 V/nm$ (d) and $D=0.4 V/nm$ (e).

Note 12: Ultra-broadband photoresponse in Dev.2.

To demonstrate the consistent performance of the TMBG transistor across a wide range of wavelength, we conduct photocurrent assessments spanning the visible, near-infrared, and sub-terahertz spectra for Dev. 2. We first characterize the device’s transport properties by measuring the two-probe resistance as a function of the top and back gate voltages (Figure S19a), revealing a gate tunable band structure illustrated in Figure S18. Subsequently, we investigate the broadband photoresponse both with and without an applied bias voltage. The device is exposed sequentially to visible (Figure S19b, e), near-infrared (Figure S19c, f), and sub-terahertz laser beams (Figure S19d, g), at a temperature of $4.5 K$. The bandgap positions determined by the transport measurements are marked by black dashed lines on the maps. Similar to Dev. 1 described in the main text (Figure 2), the zero bias photocurrent (Figure S19b-d) changes polarity around these lines, while differential photocurrent maps (Figure S19e-g) show maximized positive photocurrent at the bandgaps. For the biased device, adjusting the Fermi level into the flat band region ($\nu=0\sim4$) results in significantly pronounced negative photocurrent.

Notably, in Dev. 2, the sub-terahertz response exhibits broadened features in the photocurrent map, such as the “S”-shaped pattern in the zero bias case or peak feature in the biased device, likely due to slight variations in the twist angle across different areas of Dev. 2. Given that the sub-terahertz beam (spot size $\sim6\times6 \mathrm{mm}$) uniformly illuminates the entire TMBG area, variations in twist angle, which cause bandgap shift among different regions can contribute to the broadening of sub-terahertz response features. Detailed analysis of twist angle variations in Dev. 2 is elaborated in Note 11.


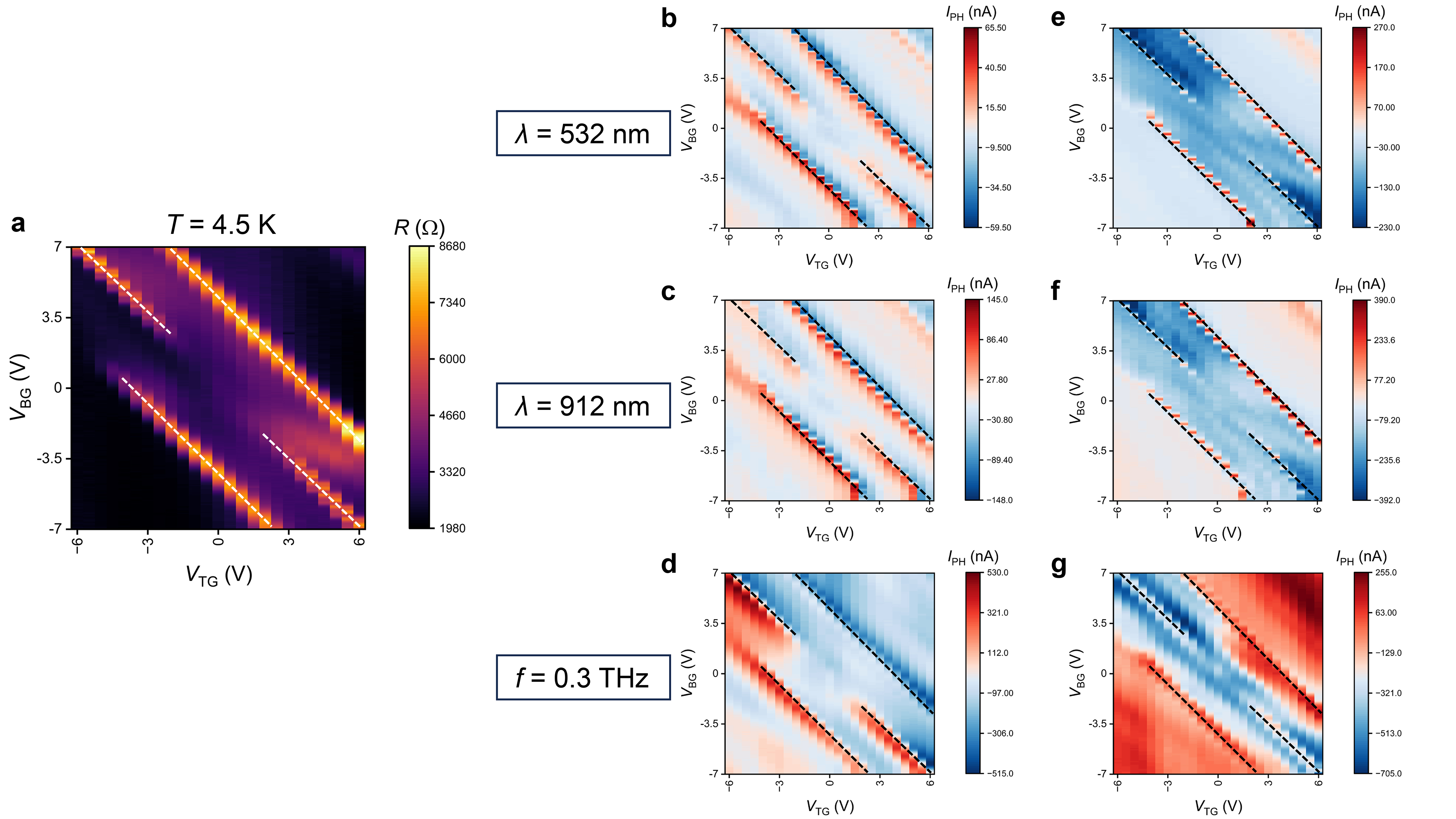


**Figure S19**

Ultra-broadband photoresponse of Dev. 2 from visible to sub-terahertz spectrum. a) Two-probe resistance map as a function of *V*_TG_ and *V*_BG_ at $T=4.5 K$. White dashed lines on the map mark the bandgaps. b-d) Photocurrent maps as a function of *V*_TG_ and *V*_BG_ at $V_{\mathrm{DS}}=0$ with light illumination at wavelengths of $532 \mathrm{nm}$ (b), $912 \mathrm{nm}$ (c), and frequency of $0.3 \mathrm{THz}$ (d). e-g) Differential photocurrent maps derived by subtracting the response at $0$ bias from that at a $30 \mathrm{mV}$ bias ($(V_{\mathrm{DS}}=30 mV)-{(V}_{\mathrm{DS}}=0)$) for the corresponding wavelengths of $532\mathrm{nm}$ (e), $912 \mathrm{nm}$ (f), and the frequency of $0.3 \mathrm{THz}$ (g). All measurements are carried out at a temperature of $4.5 K$. Black dashed lines on the maps (b-g) mark the bandgaps.

To elucidate the photocurrent generation mechanism in Dev. 2, we analyze the Seebeck coefficient term ($\frac{1}{R}\frac{dR}{dV_{G}}$) and the $TCC$, derived from the measured transport properties, as functions of *V*_BG_ and *V*_TG_, which allows us to distinguish between scenarios with and without an applied bias voltage (Figure S20). By comparing these results with experimental photocurrent maps (Figure S19), we verify that the photocurrent in the unbiased (biased) device predominately arises from the photothermoelectric (bolometric) effect, consistent with findings for Dev. 1 discussed in the main text.

In the experiment, Dev. 2 exhibits higher responsivity compared to Dev. 1, despite having lower mobility, as indicated by transport data, and more pronounced twist-angle inhomogeneity (see Figure S21). One potential explanation for this discrepancy is its smaller twist angle, which results in flatter moiré bands. In moiré-stacked graphene, the resistance due to electron-phonon scattering is known to be inversely proportional to the square of the Fermi velocity ($v_{F}$), leading to a considerable increase in $d\rho/dT$ when the Fermi level approaches the flat-band.^[10]^ As illustrated in the calculated band structures (Figure S2 and Figure S18), Dev. 2 shows a notably flatter lowest moiré band, which likely enhances the bolometric response. Furthermore, flatter energy bands contribute to sharper features in the DOS, potentially boosting the Seebeck coefficient and thereby improving the photothermoelectric effect.^[11]^ These findings suggest that adjusting the twist angle in TMBG could effectively tune the broadband photoresponse.


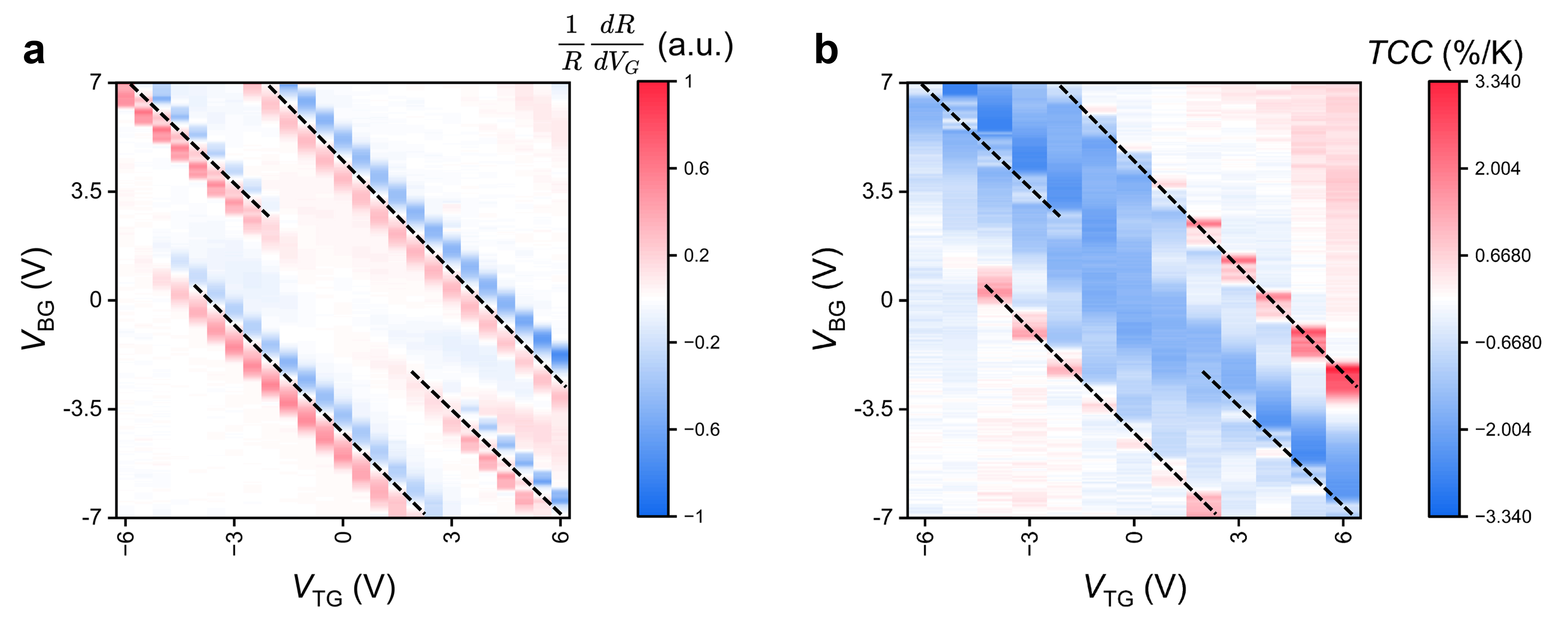


**Figure S20**

Physical mechanism of the photoresponse in Dev. 2. a) Calculated $\frac{1}{R}\frac{dR}{dV_{G}}$ as a function of *V*_TG_ and *V*_BG_, derived from the transport data using the Mott formula. b) $TCC$ calculated as a function of *V*_TG_ and *V*_BG_ at $4.5 K$, derived from temperature-dependent conductance measurements. Black dashed lines on the maps mark the bandgaps.

Note 13: Twist-angle inhomogeneity of Dev.2.

The bolometric photocurrent in biased TMBG flips the sign across the bandgap and flat moiré bands. In our experiment, a positive photocurrent peak is observed when the Fermi level aligns precisely with the bandgap induced by the superlattice. This distinctive feature, combined with SPCM, helps identify regions of the sample entering the bandgap at specific gate voltages, allowing determination of the twist angle.

Figure S21a presents the differential spatial photocurrent map of Dev. 2 at $V_{\mathrm{TG}}=5 V$ and $V_{\mathrm{BG}}=-1.7 V$, obtained by subtracting the photocurrent map at zero bias from that at a $30 \mathrm{mV}$ bias. The prominent yellow area indicates the strongest positive photoresponse near the drain electrode, suggesting that the Fermi level in this region aligns with the superlattice-induced bandgap under these conditions. To verify this, we focus the laser on this region (depicted by an orange dot in the inset of Figure S21b) and measure the differential photocurrent as a function of *V*_BG_ at $V_{\mathrm{TG}}=5 V$. A positive peak is observed at $V_{\mathrm{BG}}=-1.7 V$, corresponding to the superlattice-induced bandgap. Calculating the twist angle from the carrier density at this peak, we find it to be $\theta=1.36^{\circ}$, in agreement with the twist angle derived from transport measurements, confirming this region’s significant contribution to the device's performance.

Further analysis of the differential spatial photocurrent maps at different gate voltage configurations (Figure S21c-f) reveals a shift of the positive photocurrent peak from near the drain electrode to the source electrode. By fixing the light at the peak position indicated by SPCM (Figure S21c, e), a clear peak in differential photocurrent is observed when scanning the back gate voltage to $V_{\mathrm{BG}}=3.3 V$ and $-1.7 V$, respectively, corresponding to the superlattice-induced bandgap (for a fixed $V_{\mathrm{TG}}=0 V$). Accordingly, the twist angles for these two regions (orange dots in the insets of Figure S21d and S21f) are calculated as 1.24° and 0.89°. This suggests that lattice distortion may occur when stacking TMBG on a substrate with pre-patterned metal electrodes, leading to slight change in the twist angle between neighboring graphene layers, a phenomenon detectable through SPCM. Consequently, SPCM emerges as a promising alternative to conventional spectroscopy methods like STM^[12]^ for accurately assessing the angular alignment of the graphene moiré superlattice.


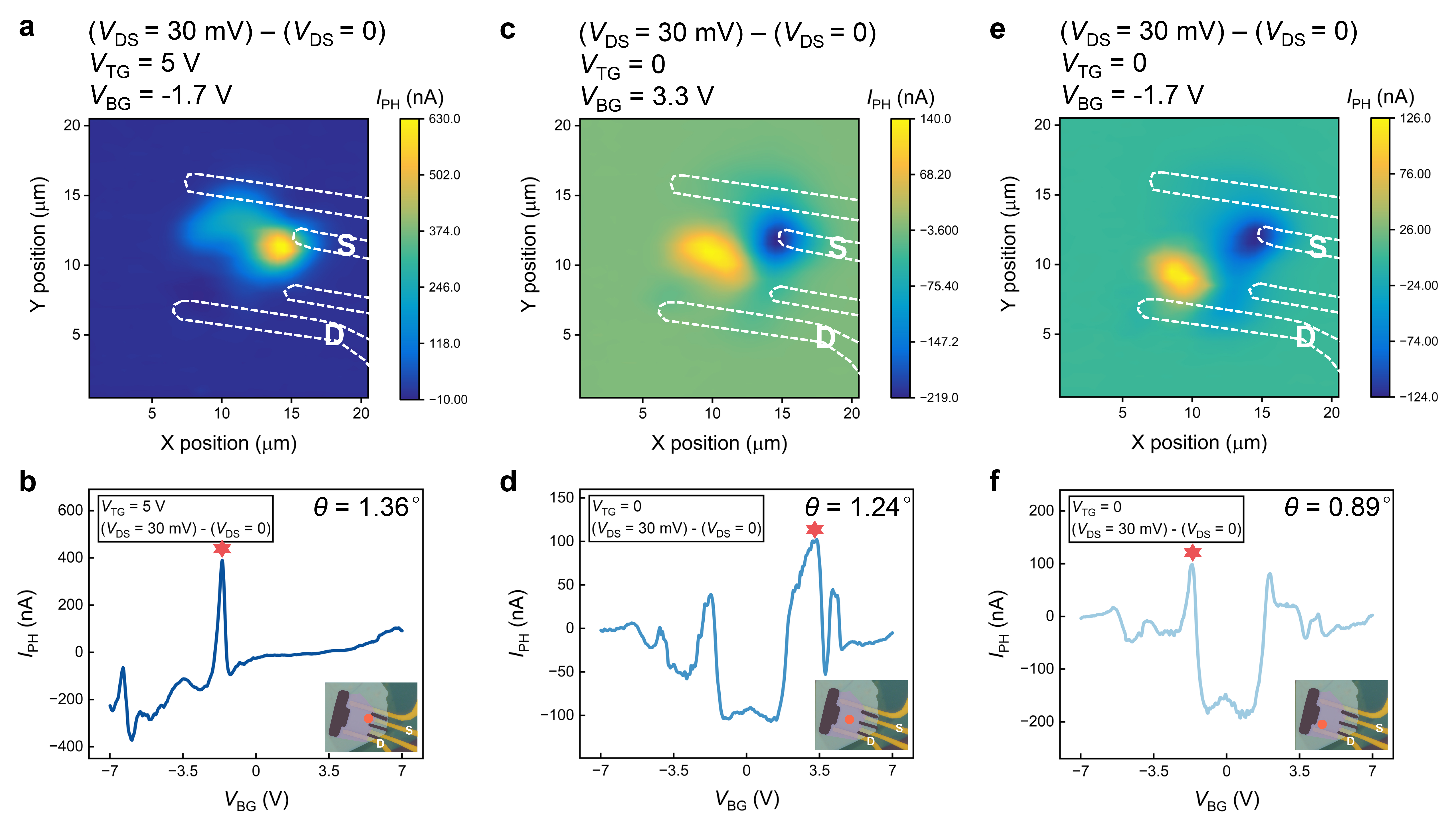


**Figure S21**

Inhomogeneity of the twist angle in Dev. 2. a,c,e) Differential SPCM at $V_{\mathrm{TG}}=5 V$, $V_{\mathrm{BG}}=-1.7 V$ (a), $V_{\mathrm{TG}}=0$, $V_{\mathrm{BG}}=3.3 V$ (c), $V_{\mathrm{TG}}=0$, $V_{\mathrm{BG}}=-1.7 V$ (e), obtained by subtracting the response at zero bias from that at a $30 \mathrm{mV}$ bias (${(V}_{\mathrm{DS}}= 30 mV)-(V_{\mathrm{DS}}=0)$). The strongest positive response is observed near the drain electrode (a), at the center (c), and near the source electrode (e), respectively. b,d,f) Photocurrent as a function of *V*_BG_ with *V*_TG_ set as $5 V$ in (b) and $0 V$ in (d, f). Inset show the optical micrograph of the device. The light is focused at the position marked with the orange dot in the inset.

Note 14: Response speed characterization.

The response speed of a photodetector is an essential performance metric for real-time and high-frequency applications. To characterize the temporal response of our TMBG device, we carried out transient photocurrent measurements under modulated sub-THz radiation at a base temperature of 4.5 K. The sub-THz beam was chopped at 5.78 kHz using a TTL-controlled modulator, and the resulting photocurrent was amplified by a low-noise current preamplifier (Stanford Research SR570, bandpass mode: 1–30 kHz) before being recorded using a high-speed oscilloscope (Keysight MSOV084A, 8 GHz bandwidth).


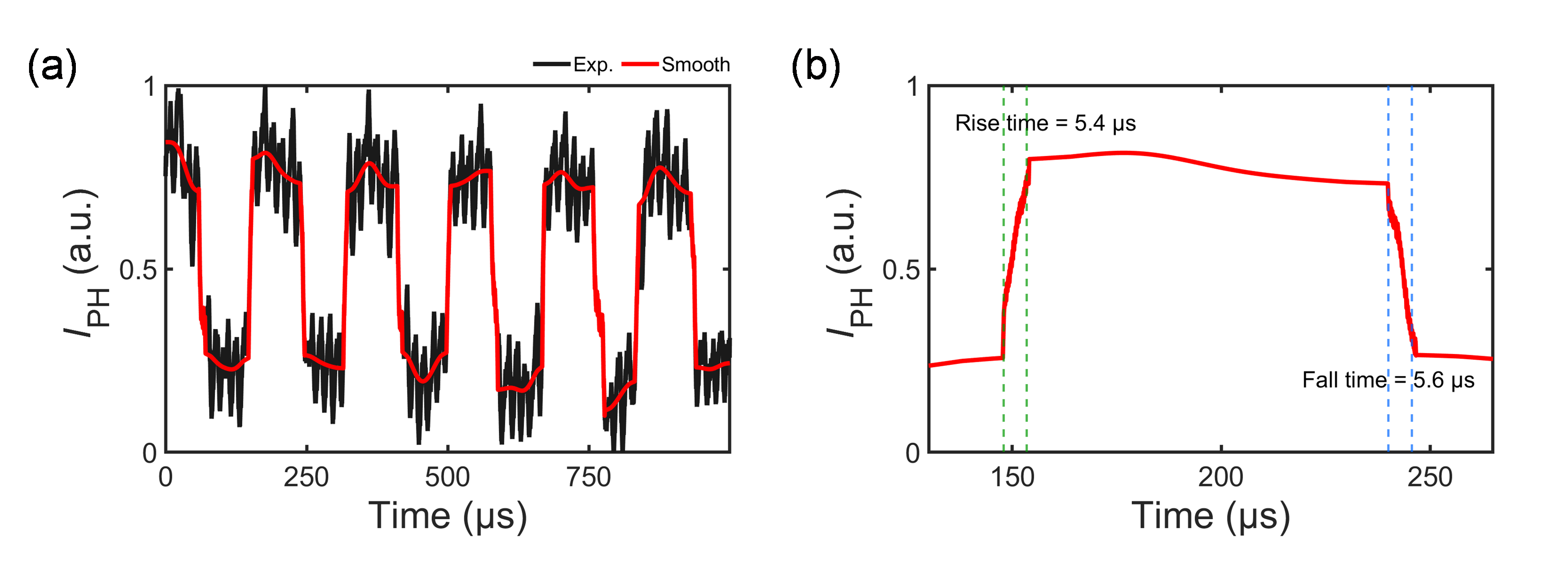


**Figure S22: Transient photoresponse characterization under sub-THz light excitation.** a) Normalized photocurrent *I*_PH_ measured as a function of time. The device is illuminated by a continuous wave laser with the frequency of 0.3 THz at 4.5 K and zero bias. The sub-THz beam is modulated by a TTL-controlled electrical chopper at a frequency of 5.78 kHz. The black curve corresponds to the experimental data, while the red curve represents smoothed data using a local averaging algorithm. b) Zoom-in plot of a single response cycle in (a), showing the rising and falling edges of the photocurrent waveform. The 10–90% rise and fall times are extracted to be 5.4 μs and 5.6 μs, respectively.

As shown in Figure S22, the transient photocurrent trace exhibits a clean, symmetric waveform with a low noise floor, indicating a robust sub-THz photoresponse. Analysis of a single modulation cycle (Figure S22b) yields 10–90% rise and fall times of 5.4 μs and 5.6 μs, respectively. While these values align with the expected bandwidth limitations of our measurement circuit, the intrinsic device response may be even faster. These results demonstrate that the TMBG photodetector exhibits a fast sub-THz response, making it viable for broadband imaging, sensing, and high-speed modulation applications.

**Table S2:** **Performance of some commercialized and graphene-based THz detectors**

| Device | Spectral  range | *R*_ex_ | NEP | Operation  temperature | Ref. |
| --- | --- | --- | --- | --- | --- |
| InSb Hot e-Bolometer | 0.06-1 THz | 4 kV/W | <800 fW/Hz^1/2^ | 4.2 K | [13] |
| InSb hot electron bolometer (QFI X) | 0.06-0.5 THz | >3.5 kV/W  (~2 A/W) | <750 fW/Hz^1/2^ | 4.2 K | [14] |
| MLG dual-gate transistor | 0.3-4.7 THz | 0.29 A/W | NA | 10 K | [15] |
| BLG dual-gate transistor | 0.13 THz | >5 A/W | 200 fW/Hz^1/2^ | 10 K | [16] |
| integrated antenna-coupled CVD graphene transistors | 0.1-1.2 THz | 59 V/W | 101 pW/Hz^1/2^ | RT | [17] |
| multilayer graphene photomixer for coherent | 0.06-0.7 THz | 0.1 µA/W | 162 pW/Hz^1/2^ | 300 K | [18] |
| Weyl-semimetal (NbIrTe_4_) and graphene heterojunction | 0.02-0.3 THz | 264.6 V/W | 280 pW/Hz^1/2^ | RT | [19] |
| TMBG dual-gate transistor | 0.3 THz-visible | 16.91 A/W  51 mA/W | 27 fW/Hz^1/2^  47 pW/Hz^1/2^ | 4.5 K  280 K | this work |

Note: NA: not available

**Table S3. Performance of terahertz photodetectors based on other semimetals and low-dimensional material systems.**

| Device | Spectral  range | *R*_ex_ | NEP | Operation  temperature | Ref. |
| --- | --- | --- | --- | --- | --- |
| TaFe_1.25_Te_3_ | 0.1 THz | 7.58 V/W | 156 pW/Hz^1/2^ | RT | [20] |
| PdSe_2_ | 0.3 THz-visible | 5 mA/W | 900 pW/Hz^1/2^ | RT | [21] |
| Ta_2_NiSe_5_ | 0.03-0.3 THz | 36 mA/W | 417 pW/Hz^1/2^ | RT | [22] |
| Graphene/NiTeSe | 0.12-0.3 THz | 19 mA/W | 1.17 nW/Hz^1/2^ | RT | [23] |
| NbSe_2_ | 0.1-1.2 THz | 0.57 A/W | 130 pW/Hz^1/2^ | RT | [24] |
| NbP | 0.04-0.3 THz | 40.5 V/W | 200 pW/Hz^1/2^ | RT | [25] |
| TaIrTe_4_ | 0.1 THz | 13.7 A/W  0.3 A/W | 50 fW/Hz^1/2^  1 pW/Hz^1/2^ | 4 K  300 K | [26] |
| suspended NbN/Nb_5_N_6_ | 0.65 THz | NA | 420 fW/Hz^1/2^ | 5 K | [27] |
| NbFeTe_2_ | 0.1-0.3 THz | 2.21 V/W | 338 pW/Hz^1/2^ | RT | [28] |
| Nb_3_Se_12_I | 0.3 THz -ultraviolet | ~0.7 V/W | 2120 pW/Hz^1/2^ | RT | [29] |
| T_d_-MoTe_2_ | 0.53 THz- ultraviolet | 0.53 mA/W | 2700 pW/Hz^1/2^ | RT | [30] |
| TMBG dual-gate transistor | 0.3 THz-visible | 16.91 A/W  51 mA/W | 27 fW/Hz^1/2^  47 pW/Hz^1/2^ | 4.5 K  280 K | this work |

(If multiple performance metrics at different wavelengths were reported in the reference, the one closest to 0.3 THz was selected for comparison.)

References

1. R. Bistritzer, and A. H. MacDonald, "Moire Bands in Twisted Double-Layer Graphene," *Proceedings of the National Academy of Sciences of the United States of America* 108, no. 30 (2011): 12233. https://doi.org/10.1073/pnas.1108174108

2. J. Y. Lee, E. Khalaf, [S. Liu](https://www.nature.com/articles/s41467-019-12981-1#auth-Shang-Liu-Aff1), et al., "Theory of Correlated Insulating Behaviour and Spin-Triplet Superconductivity in Twisted Double Bilayer Graphene," *Nature Communications* 10, no. 1 (2019): 5333. https://doi.org/10.1038/s41467-019-12981-1

3. J. Liu, Z. Ma, J. Gao, and X. Dai, "Quantum Valley Hall Effect, Orbital Magnetism, and Anomalous Hall Effect in Twisted Multilayer Graphene Systems," *Physical Review X* 9, no. 3 (2019): 031021. https://doi.org/10.1103/PhysRevX.9.031021

4. J. Jung, and A. H. MacDonald, "Accurate Tight-Binding Models for the π Bands of Bilayer Graphene," *Physical Review B* 89, no. 3 (2014): 035405. https://doi.org/10.1103/PhysRevB.89.035405

5. S. Chen, M. He, Y.-H. Zhang, et al., "Electrically Tunable Correlated and Topological States in Twisted Monolayer–Bilayer Graphene," *Nature Physics* 17, no. 3 (2020): 374. https://doi.org/10.1038/s41567-020-01062-6

6. J. Park, Y. H. Ahn, and C. Ruiz-Vargas, "Imaging of Photocurrent Generation and Collection in Single-Layer Graphene," *Nano Letters* 9, no. 5 (2009): 1742. https://doi.org/10.1021/nl8029493

7. F. N. Xia, T. Mueller, R. Golizadeh-Mojarad, et al., "Photocurrent Imaging and Efficient Photon Detection in a Graphene Transistor," *Nano Letters* 9, no. 3 (2009): 1039. https://doi.org/10.1021/nl8033812

8. M. Buscema, M. Barkelid, V. Zwiller, H. S. J. van der Zant, G. A. Steele, and A. Castellanos-Gomez, "Large and Tunable Photothermoelectric Effect in Single-Layer MoS_2_," *Nano Letters* 13, no. 2 (2013): 358. https://doi.org/10.1021/nl303321g

9. O. Çakıroğlu, N. Mehmood, M. M. Çiçek, A. A. Karluk, H. R. Rasouli, E. Durgun, and T. S. Kasırga, "Thermal Conductivity Measurements in Nanosheets via Bolometric Effect," *2D Materials* 7, no. 3 (2020): 035003. https://doi.org/10.1088/2053-1583/ab8048

10. H. Polshyn, M. Yankowitz, S. Chen, et al., "Large Linear-in-Temperature Resistivity in Twisted Bilayer Graphene," *Nature Physics* 15, no. 10 (2019): 1011. https://doi.org/10.1038/s41567-019-0596-3

11. A. Kommini, and Z. Aksamija, "Very High Thermoelectric Power Factor Near Magic Angle in Twisted Bilayer Graphene," *2D Materials* 8, no. 4 (2021): 045022. https://doi.org/10.1088/2053-1583/ac161d

12. H. Kim, Y. Choi, C. Lewandowski, et al., "Evidence for Unconventional Superconductivity in Twisted Trilayer Graphene," *Nature* 606, no. 7914 (2022): 494. https://doi.org/10.1038/s41586-022-04715-z

13. *Bolometer Systems* (IRLabs). https://www.irlabs.com/products/bolometers/bolometer-systems/

14. *InSb hot electron bolometer* (QMC Instruments). https://www.qmcinstruments.co.uk/insb-hot-electron-bolometer

15. J. M. Caridad, O. Castello, S. M. López Baptista, et al., "Room-Temperature Plasmon-Assisted Resonant THz Detection in Single-Layer Graphene Transistors," *Nano Letters* 24, no. 3 (2024): 935. https://doi.org/10.1021/acs.nanolett.3c04300

16. I. Gayduchenko, S. G. Xu, G. Alymov, et al., "Tunnel Field-Effect Transistors for Sensitive Terahertz Detection," *Nature Communications* 12, no. 1 (2021): 543. https://doi.org/10.1038/s41467-020-20721-z

17. F. Ludwig, A. Generalov, J. Holstein, et al., "Terahertz Detection with Graphene FETs: Photothermoelectric and Resistive Self-Mixing Contributions to the Detector Response," *ACS Applied Electronic Materials* 6, no. 4 (2024): 2197. https://doi.org/10.1021/acsaelm.3c01511

18. M. D. Thomson, F. Ludwig, J. Holstein, R. Al-Mudhafar, S. Al-Daffaie, and H. G. Roskos, "Coherent Terahertz Detection via Ultrafast Dynamics of Hot Dirac Fermions in Graphene," *ACS Nano* 18, no. 6 (2024): 4765. https://doi.org/10.1021/acsnano.3c08731

19. Y. He, L. Yang, Z. Hu, et al., "Selective Growth of Type‐II Weyl‐Semimetal and Van der Waals Stacking for Sensitive Terahertz Photodetection," *Advanced Functional Materials* 34, no. 12 (2023): 2311008. https://doi.org/10.1002/adfm.202311008

20. D. Zhang, S. Ni, C. Liu, et al., "Zero-Bias Uncooled Broadband Terahertz Detection with a TaFe₁.₂₅Te₃/Graphene Heterostructure for Imaging and Communication," *Advanced Optical Materials* 13, no. 21 (2025): 2500748. https://doi.org/10.1002/adom.202500748

21. Z. Dong, W. Yu, L. Zhang, et al., "Highly Efficient, Ultrabroad PdSe₂ Phototransistors from Visible to Terahertz Driven by Multiphysical Mechanism," *ACS Nano* 15, no. 12 (2021): 20403. https://doi.org/10.1021/acsnano.1c08756

22. Z. Dong, W. Guo, L. Zhang, et al., "Excitonic Insulator Enabled Ultrasensitive Terahertz Photodetection with Efficient Low-Energy Photon Harvesting," *Advanced Science* 9, no. 36 (2022): 2204580. https://doi.org/10.1002/advs.202204580

23. K. Zhang, Z. Hu, L. Zhang, et al., "Ultrasensitive Self-Driven Terahertz Photodetectors Based on Low-Energy Type-II Dirac Fermions and Related Van der Waals Heterojunctions," *Small* 19, no. 1 (2023): 2205329. https://doi.org/10.1002/smll.202205329

24. M. Jiang, H. Xing, L. Zhang, et al., "Fast Switching of Bolometric and Self-Powered Effects in 2H-NbSe₂ for High-Efficiency Low-Energy Photon Harvesting," *Advanced Optical Materials* 11, no. 12 (2023): 2300074. https://doi.org/10.1002/adom.202300074

25. Z. Hu, B. Zhao, Y. Wei, et al., "High-Performance Terahertz Detection in Weyl Semimetal NbP Nanosheets," *Infrared Physics & Technology* 149 (2025): 105896. https://doi.org/10.1016/j.infrared.2025.105896

26. T. Xi, H. Jiang, J. Li, et al., "Terahertz Sensing Based on the Nonlinear Electrodynamics of the Two-Dimensional Correlated Topological Semimetal TaIrTe₄," *Nature Electronics* 8 (2025): 578. https://doi.org/10.1038/s41928-025-01397-z

27. H. Shi, T. Xu, Y. Zhe, et al., "A Niobium Nitride Superconducting Hot Electron Bolometer Direct Terahertz Detector Working at 9 K," *Applied Physics Letters* 126, no. 4 (2025): 042601. https://doi.org/10.1063/5.0231315

28. D. Wang, L. Yang, Z. Hu, et al., "Antiferromagnetic Semimetal Terahertz Photodetectors Enhanced through Weak Localization," *Nature Communications* 16 (2025): 25. https://doi.org/10.1038/s41467-024-55426-0

29. J. Zhang, Z. Hu, Q. Yang, et al., "Strong Anisotropy and Giant Photothermoelectricity of 1D Alloy Nb₃Se₁₂I-Based Photodetector for Ultrabroadband Light-Detection and Encryption Imaging Application," *Advanced Materials* 37, no. 1 (2025): 2410275. https://doi.org/10.1002/adma.202410275

30. Q. Yang, X. Wang, Z. He, et al., "A Centimeter-Scale Type-II Weyl Semimetal for Flexible and Fast Ultra-Broadband Photodetection from Ultraviolet to Sub-Millimeter Wave Regime," *Advanced Science* 10, no. 17 (2023): 2205609. https://doi.org/10.1002/advs.202205609
